# Multiscale Mathematical Modeling in Dental Tissue Engineering: Toward Computer-Aided Design of a Regenerative System Based on Hydroxyapatite Granules, Focussing on Early and Mid-Term Stiffness Recovery

**DOI:** 10.3389/fphys.2016.00383

**Published:** 2016-09-21

**Authors:** Stefan Scheiner, Vladimir S. Komlev, Alexey N. Gurin, Christian Hellmich

**Affiliations:** ^1^Institute for Mechanics of Materials and Structures, Department of Civil Engineering, TU Wien—Vienna University of TechnologyVienna, Austria; ^2^A.A. Baikov Institute of Metallurgy and Materials Science, Russian Academy of SciencesMoscow, Russia; ^3^Institute of Laser and Information Technologies, Russian Academy of SciencesMoscow, Russia; ^4^Central Scientific Research Institute of Dentistry and Maxillofacial SurgeryMoscow, Russia

**Keywords:** homogenization, multiscale, hydroxyapatite, material optimization, bone ingrowth

## Abstract

We here explore for the very first time how an advanced multiscale mathematical modeling approach may support the design of a provenly successful tissue engineering concept for mandibular bone. The latter employs double-porous, potentially cracked, single millimeter-sized granules packed into an overall conglomerate-type scaffold material, which is then gradually penetrated and partially replaced by newly grown bone tissue. During this process, the newly developing scaffold-bone compound needs to attain the stiffness of mandibular bone under normal physiological conditions. In this context, the question arises how the compound stiffness is driven by the key design parameters of the tissue engineering system: macroporosity, crack density, as well as scaffold resorption/bone formation rates. We here tackle this question by combining the latest state-of-the-art mathematical modeling techniques in the field of multiscale micromechanics, into an unprecedented suite of highly efficient, semi-analytically defined computation steps resolving several levels of hierarchical organization, from the millimeter- down to the nanometer-scale. This includes several types of homogenization schemes, namely such for porous polycrystals with elongated solid elements, for cracked matrix-inclusion composites, as well as for assemblies of coated spherical compounds. Together with the experimentally known stiffnesses of hydroxyapatite crystals and mandibular bone tissue, the new mathematical model suggests that early stiffness recovery (i.e., within several weeks) requires total avoidance of microcracks in the hydroxyapatite scaffolds, while mid-term stiffness recovery (i.e., within several months) is additionally promoted by provision of small granule sizes, in combination with high bone formation and low scaffold resorption rates.

## 1. Introduction

The importance of mathematical modeling in dentistry and related fields has steadily increased over the last decades. Thereby, the most popular examples concern Finite Element models of the mandibular system, dating back to at least the early 1990s (Hart et al., 1992; Koritoth and Versluis, 1997). While these models have been continuously improved over recent years, the proper choice of mechanical material properties as key model input parameters has gained particular attention. It has become more and more customary to derive these parameters directly from computed tomography (CT) images of the investigated organs, be it through a more heuristic, regression analysis-based approach (van Ruijven et al., 2007), or based on the combination of X-ray physics and continuum micromechanics concepts (Hellmich et al., 2008). The latter approach explicitly considers the hierarchical organization of bone down to the microscopic scales of cellular activity, i.e., to those which are in the very focus of modern bioengineering approaches, including regenerative medicine strategies. In the present contribution, we wish to extend the application range of dental mathematical modeling, from the mechanics of standard mandibular systems, to latest developments in modern bioengineering approaches. In more detail, we here explore for the very first time how an advanced multiscale mathematical modeling approach may support the design of a provenly successful tissue engineering concept for regenerating large bone defects in the human mandible (Komlev et al., 2002, 2003). The latter concept employs double-porous, potentially cracked, single millimeter-sized granules packed into an overall scaffold material, which is then gradually penetrated and partially replaced by newly grown bone tissue.

**Figure 1:**
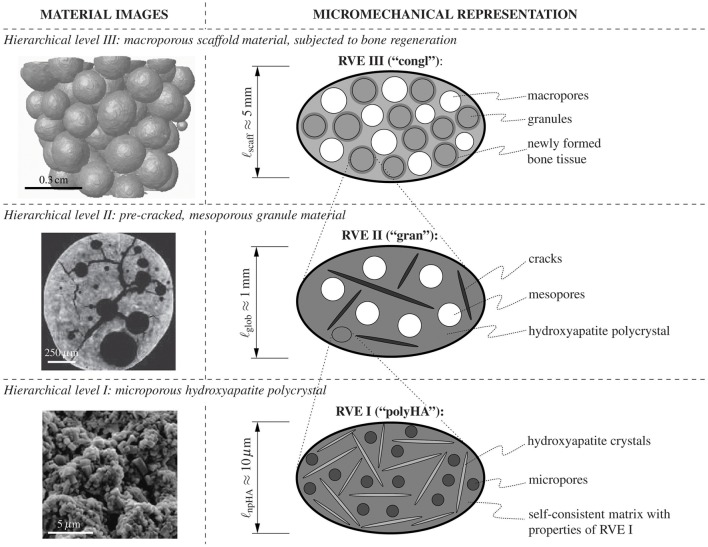
**Investigated material system**. Three-level representation of the hydroxyapatite-based granular biomaterial (column on the right-hand side), following the morphological features found in images on different observation scales (column on the left-hand side); the depicted images have been acquired by means of scanning electron microscopy (hierarchical level I) and μCT imaging techniques (hierarchical levels II and III).

The granules themselves, exhibiting diameters from a few hundred micrometers to one or two millimeters, result from a processing route based on the effect of immiscible fluids (Komlev et al., 2002, 2003). Key morphological features of these granules are seen in the left-hand column of Figure 1: Firstly, they contain pores of two different characteristic lengths: small pores, with a characteristic length of one to a few micrometers—these pores are termed “micropores” hereafter; and large pores, with a characteristic length of several hundred micrometers—these pores are termed “mesopores” hereafter. A composite of randomly oriented hydroxyapatite crystals and the micropores constitutes the “base material” making up the granules. Upon increasing the observation scale by several orders of magnitude, one can discern not only the aforementioned mesopores, but also cracks which pervade the individual granules. Finally, the scaffold material is made up of the above described granules, with pore space in-between—due to the characteristic length of these pores, which is of the order of the granule diameters, these pores are termed “macropores” throughout the present paper. It is in these macropores, where the regeneration process starts, i.e., where new bone tissue is formed after implantation of the scaffold systems. During this regeneration process, the newly developing scaffold-bone compound needs to attain the stiffness of mandibular bone under normal physiological conditions. In this context, the question arises how the compound stiffness is driven by the key design parameters of the tissue engineering system: macroporosity, crack density, as well as scaffold resorption/bone formation rates. We here tackle this question by combining the latest state-of-the-art mathematical modeling techniques in the field of multiscale micromechanics, as reviewed in Section 2.1, into an unprecedented suite of highly efficient semi-analytically defined computation steps resolving several levels of hierarchical organization, from the millimeter- down to the nanometer-scale. This includes several types of homogenization schemes, namely such for porous polycrystals with elongated solid elements, for cracked matrix-inclusion composites, as well as for assemblies of coated spherical compounds; described in great detail in Sections 2.2–2.5. These mathematical developments allow for first-ever insights into the mechanical functioning of the investigated tissue engineering system, including the role the aforementioned design parameters, as is documented in Section 3, before the paper finds its conclusion in Section 4.

## 2. Methods – mathematical modeling in the framework of multiscale continuum micromechanics, with corresponding animal models and biomaterial experiments

### 2.1. Consideration of material hierarchy – introduction of representative volume elements (RVEs)

In recent years, continuum micromechanics-based homogenization theory (Hill, 1963; Suquet, 1997; Zaoui, 1997, 2002) turned out as particularly suitable means for the mathematical modeling of the mechanical behavior of complex hierarchical material systems found in biology and biomedical engineering (Hellmich and Ulm, 2002; Hellmich et al., 2004a; Fritsch and Hellmich, 2007; Bertrand and Hellmich, 2009; Fritsch et al., 2009; Hamed et al., 2010; Fritsch et al., 2013; Scheiner et al., 2016). In this context, a material is understood as a micro-heterogeneous body filling a macro-homogeneous representative volume element (RVE) with characteristic length ℓ, ℓ ≫ *d*, *d* standing for the characteristic length of inhomogeneities within the RVE, and ℓ ≪ L, L standing for the characteristic lengths of geometry or loading of a structure built up by the material defined on the RVE. Notably, for achieving results characterized by a quite good accuracy of about 5%, *d* and ℓ need to be separated by not more than a factor of 2 (Drugan and Willis, 1996), while ℓ and L need to be separated by a factor of 5 to 50 (Kohlhauser and Hellmich, 2013).

In general, the microstructure within such an RVE is so complicated that it cannot be described in complete detail. Therefore, the microstructural description within an RVE is restricted to the choice of quasi-homogeneous subdomains (called material phases), which are characterized by the following properties: (i) their shapes, (ii) their volume fractions within the RVE, (iii) their mechanical properties, and (iv) their mechanical interactions. Based on these characteristics, one can then derive the homogenized (upscaled) behavior of the material on the observation scale of the RVE, i.e., the relation between homogeneous deformations acting on the boundary of the RVE and resulting macroscopic (average) stresses. If a single material phase is micro-heterogeneous itself, its mechanical behavior can be estimated by means of introducing RVEs within this phase, with characteristic lengths ℓ_1_ ≤ *d*, comprising again inhomogeneities with characteristic length *d*_1_ ≪ ℓ_1_, and so on. Such an approach is referred to as multi-step homogenization. At sufficiently low observation scales, it may provide “universal” phase properties, i.e., properties which are invariant throughout an entire material class, such as all bone tissues occurring in vertebrates (Fritsch and Hellmich, 2007).

For the material system investigated herein, i.e., for assemblies of double-porous hydroxyapatite granules with bone tissue optionally coating the granules, the following suite of RVEs, with increasing characteristic lengths, is introduced:

- On *hierarchical level I*, a microporous, overall isotropic, hydroxyapatite polycrystal emerges, see the bottom of Figure 1, showing a scanning electron micrograph of the polycrystalline microstructure on the left-hand side, and a two-dimensional schematical representation of the (actually three-dimensional) RVE I on the right-hand side: The latter is composed of spherical micropores (with volume fraction $\phi_{\text{micro}}^{\text{polyHA}}$, also called polycrystalline microporosity). These micropores interact mutually with randomly oriented cylindrical hydroxyapatite crystals, with volume fraction $f_{\text{HA}}^{\text{polyHA}}=1-\phi_{\text{micro}}^{\text{polyHA}}$. The microporosity typically amounts to $\phi_{\text{micro}}^{\text{polyHA}}=0.445$ (Dejaco et al., 2012). The characteristic length of the polycrystalline RVE I is in the order of 10 μm, see the bottom line of Figure 1.
- On *hierarchical level II*, a mesoporous, cracked matrix material makes up the individual granules, see the center of Figure 1, showing a micro-computed tomography (μCT) image of the microstructure within a granule on the left-hand side, and a two-dimensional schematical representation of the (actually three-dimensional) RVE II on the right-hand side: Namely, penny-shaped cracks, with vanishing volume fraction, while being quantified in number and size through the crack density parameter ϵ, see Equation (2), and spherical mesopores, with volume fraction $\phi_{\text{meso}}^{\text{gran}}$, are embedded in the polycrystal matrix with properties arising from the structure of RVE I. Within RVE II, the latter matrix fills the volume fraction $f_{\text{polyHA}}^{\text{gran}}=1-\phi_{\text{meso}}^{\text{gran}}$. The mesoporosity typically amounts to $\phi_{\text{meso}}^{\text{gran}}=0.189$ (Dejaco et al., 2012). The characteristic length of RVE II is in the order of 1mm, see the middle row in Figure 1.
- On *hierarchical level III*, a macroporous conglomerate material consisting of mesoporous, cracked hydroxapatite granules and newly grown bone tissue emerges, see the top of Figure 1: granules with the stiffness of RVE II described above and filling volume fraction $f_{\text{gran}}^{\text{congl}}$, are surrounded by layers of newly grown bone tissue, with volume fraction $f_{\text{bone}}^{\text{congl}}$ and stiffness derived from the ultrasonic tests of Ashman and van Buskirk (1987). These coated spherical elements are assembled, in mutual contact, to a granular conglomerate with macropores, with volume fraction $\phi_{\text{macro}}^{\text{congl}}$, in-between. At the time of granule implantation, no bone tissue has been formed yet, and this initial configuration is characterized by $f_{\text{bone}}^{\text{congl}}=0$.

### 2.2. Stiffness tensor homogenization at hierarchical level i: elasticity of porous hydroxyapatite polycrystal

The elastic behavior of interpenetrating non-spherical crystals with pores in-between can be particularly well represented by the self-consistent stiffness estimate introduced by Fritsch et al. (2006), where infinitely many solid phases oriented in all space directions as well as one spherical pore phase are embedded in a matrix with zero volume fraction and with the stiffness of the homogenized material itself. The corresponding stiffness tensor of the water-filled porous polycrystal at hierarchical level I of Figure 1 reads as

(1) $$\begin{array}{l} {\mathbb{C}}_{\text{polyHA}}=\{f_{\text{HA}}^{\text{polyHA}}{\mathbb{C}}_{\text{HA}}:\left[\overset{2\pi }{\underset{\varphi \;=\;0}{\int }}\overset{\pi }{\underset{\vartheta \;=\;0}{\int }}{\left[𝕀+{\mathbb{P}}_{\text{cyl}}^{\text{polyHA}}(\vartheta ,\varphi ):\left({\mathbb{C}}_{\text{HA}}-{\mathbb{C}}_{\text{polyHA}}\right)\right]}^{-1}\frac{\text{sin}\vartheta \text{ d}\vartheta \text{ d}\varphi }{4\pi }\right]\\ \;+\;\phi_{\text{micro}}^{\text{polyHA}}{\mathbb{C}}_{\text{micro}\phi }:{\left[𝕀-{\mathbb{P}}_{\text{sph}}^{\text{polyHA}}:\left({\mathbb{C}}_{\text{micro}\phi }-{\mathbb{C}}_{\text{polyHA}}\right)\right]}^{-1}\}\\ \;:\{f_{\text{HA}}^{\text{polyHA}}\left[\overset{2\pi }{\underset{\varphi \;=\;0}{\int }}\overset{\pi }{\underset{\vartheta \;=\;0}{\int }}{\left[𝕀+{\mathbb{P}}_{\text{cyl}}^{\text{polyHA}}(\vartheta ,\varphi ):\left({\mathbb{C}}_{\text{HA}}-{\mathbb{C}}_{\text{polyHA}}\right)\right]}^{-1}\frac{\text{sin}\vartheta \text{ d}\vartheta \text{ d}\varphi }{4\pi }\right]\\ \;+{\;\phi_{\text{micro}}^{\text{polyHA}}{\left[𝕀-{\mathbb{P}}_{\text{sph}}^{\text{polyHA}}:\left({\mathbb{C}}_{\text{micro}\phi }-{\mathbb{C}}_{\text{polyHA}}\right)\right]}^{-1}\}}^{-1}, \end{array}$$

where $\phi_{\text{micro}}^{\text{polyHA}}$ and $f_{\text{HA}}^{\text{polyHA}}$ are the volume fractions of the micropores and the hydroxyapatite needles; ℂ_HA_ and ℂ_microϕ_, respectively, are the fourth-order stiffness tensors of the hydroxyapatite crystals and of the micropores, respectively; ϑ and φ are the Euler angles quantifying the orientations of the hydroxyapatite crystals; ${\mathbb{P}}_{\text{cyl}}^{\text{polyHA}}\left(\vartheta ,\varphi \right)$ and ${\mathbb{P}}_{\text{sph}}^{\text{polyHA}}$, respectively, are the fourth-order Hill (or morphology) tensors related to cylindrical and spherical inclusions, respectively, embedded in a matrix made up of the microporous hydroxyapatite polycrystal; and *I* is the fourth-order unit tensor, the components of which are defined via the Kronecker delta δ_*ij*_ (δ_*ij*_ = 1 if *i* = *j* and δ_*ij*_ = 0 if *i*≠1), namely *I*_*ijkl*_ = 1/2(δ_*ik*_ δ_*jl*_ + δ_*il*_δ_*jk*_). The double integrals in Equation (1) can be evaluated based on Stroud's integration equations (Stroud, 1971; Pichler et al., 2009). Details regarding the computation of the Hill tensors ${\mathbb{P}}_{\text{cyl}}^{\text{polyHA}}\left(\vartheta ,\varphi \right)$ and ${\mathbb{P}}_{\text{sph}}^{\text{polyHA}}$ are presented in the Supplementary Material of this paper.

Numerical evaluation of Equation (1) requires knowledge of stiffness tensors ℂ_HA_ and ℂ_microϕ_. The isotropic stiffness of hydroxyapatite is known from the experiments performed by Katz and co-workers (Katz and Ukraincik, 1971; Gilmore and Katz, 1982), yielding a Young's modulus of *E*_HA_ = 114 GPa, and a Poisson's ratio of ν_HA_ = 0.27, see also (Hellmich and Ulm, 2002; Hellmich et al., 2004b). We here consider the case where the pore fluid is free to leave the microporosity upon loading of RVE I. This relates to so-called drained conditions, with ℂ_microϕ_ = 0 (Thompson and Willis, 1991).

### 2.3. Stiffness tensor homogenization at hierarchical level ii: elasticity of cracked mesoporous granule material

Matrix-inclusion composites are preferentially represented by a so-called Mori-Tanaka-type morphology (Mori and Tanaka, 1973; Benveniste, 1987). At hierarchical level II, two types of inclusions are embedded into a matrix made of the porous polycrystal with a stiffness resulting from the homogenization scheme of Section 2.2: (i) spherical pores (the volume fraction of which is the mesoporosity $\phi_{\text{meso}}^{\text{gran}}$), and (ii) penny-shaped (non-frictional, open) cracks oriented in all space directions. The latter fill an only negligible volume fraction, so that their amount is quantified through their number per volume *N* and their radius *r*_cr_, combined into the so-called crack density parameter according to Budianksy and O'Connell (1976):

(2) $$\begin{array}{l} \epsilon =Nr_{\text{cr}}^{3}. \end{array}$$

Based on the works of Deudé et al. (2002) and Dormieux et al. (2004), the corresponding stiffness estimate of the mesoporous, pre-cracked granule material reads as 
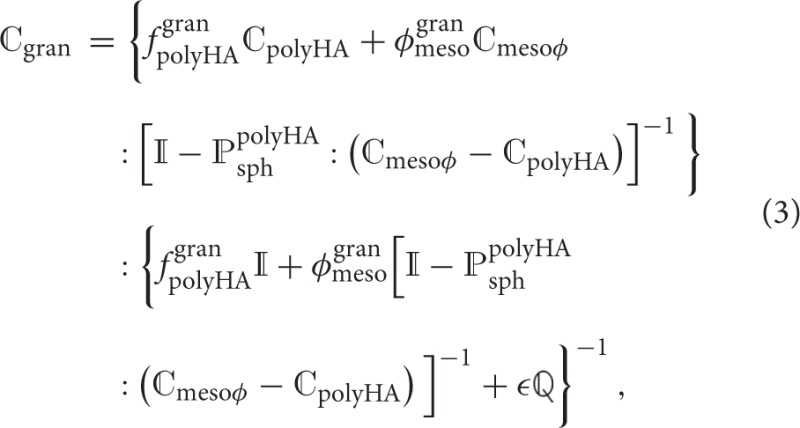
 where $f_{\text{polyHA}}^{\text{gran}}$ and $\phi_{\text{meso}}^{\text{gran}}$ are the volume fractions of the matrix made up of the microporous hydroxyapatite polycrystal and of the mesopores; ℂ_polyHA_ is the fourth-order stiffness tensor of the microporous hydroxyapatite polycrystal matrix (see Section 2.2); ℂ_mesoϕ_ is the fourth-order stiffness tensor of the mesopores, defined analogously to the stiffness tensor of the micropores (i.e., drained), and ${\mathbb{P}}_{\text{sph}}^{\text{polyHA}}$ is the Hill tensor for spherical inclusions embedded in the isotropic microporous hydroxyapatite polycrystal matrix, see the Supplementary Material for details. Tensor 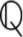, also occurring in Equation (3), is defined via the Poisson's ratio of the microporous hydroxyapatite polycrystal, ν_polyHA_, 
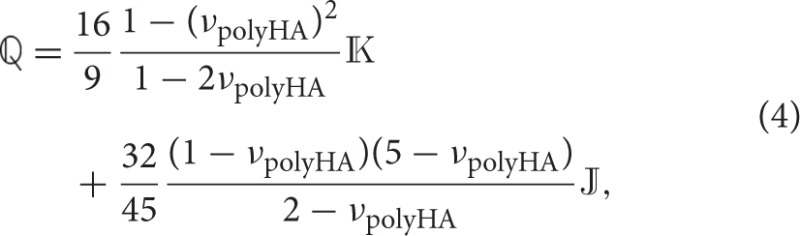
 see (Dormieux et al., 2004).

### 2.4. Microstress and microstrain fields in bone tissue-coated hydroxyapatite granules and in the macropores – matrix-inclusion problems of the Hervé-Zaoui and of the eshelby type

**Figure 2:**
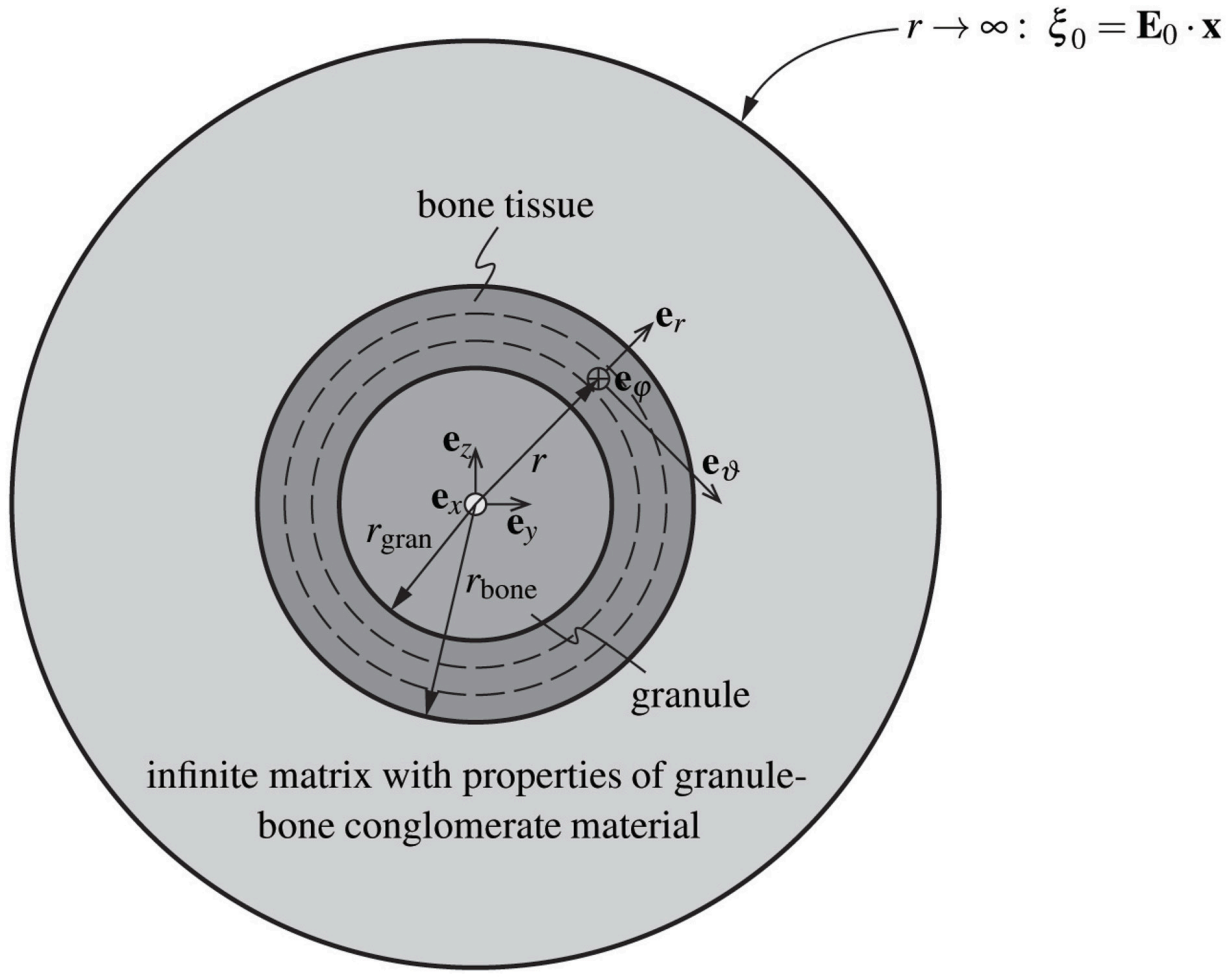
**Adaption of the model of Hervé and Zaoui (1993)**. Homogenization of the stiffness of the macro-porous granular scaffold material considering bone ingrowth, with spherical granules coated by bone tissue, and embedded in a polycrystal-type composite material consisting of the bone-coated granules and macropores.

Having reviewed and confirmed, respectively, the reliability of advanced self-consistent and Mori-Tanaka homogenization schemes at hierarchical level I (in Section 2.2) and at hierarchical level II (in Section 2.3), we now assign corresponding stiffness properties to individual granules, which we surround by increasingly thick coatings of newly formed bone tissue, and which we then pile up to a conglomerate of bone tissue-coated granules, see the granule and bone phases in RVE III shown in Figure 1. This goes beyond extending the classical self-consistent and Mori-Tanaka estimates, but requires the consideration of shell-like morphologies as pioneered by Hervé and Zaoui (1993); and this adaption motivates the following further developments: While the phase strains at hierarchical levels I and II were all estimated from homogenous inclusion strains of the underlying Eshelby-problem consisting of a spherical or cylindrical inclusion being embedded either into a real matrix, or into a fictitious matrix with the stiffness of the homogenized material, only the macropore phase exhibits this homogeneous property as concerns the RVE of hierarchical level III. In turn, as for the coated granule phase, Hervé-Zaoui's matrix-*coated* inclusion problem is considered (see Figure 2). This problem is characterized by the following boundary and field conditions:

- Homogeneous strains at any location which is infinitely far from the inclusion center:
  (5) $$\begin{array}{l} r\to \infty :\;\xi \to {\text{E}}_{0}\cdot \text{x}, \end{array}$$
  where **x** is the position vector, **ξ** is the displacement field, and **E**_0_ is the field of uniform displacements to which the auxiliary matrix with the properties of the overall conglomerate is subjected infinitely far from the inclusion.
- Equilibrium condition:
  (6) $$\begin{array}{l} 0\leq r<\infty :\text{ div}\text{σ}=0, \end{array}$$
  where div denotes the divergence operator. Evaluation of Equation (6) yields, when considering spherical coordinates, the following three differential equations (Salençon, 2001):
  (7) $$\begin{array}{r} \frac{\partial \sigma_{rr}}{\partial r}+\frac{1}{r}\frac{\partial \sigma_{r\vartheta }}{\partial \vartheta }+\frac{1}{r\text{sin}\vartheta }\frac{\partial \sigma_{r\varphi }}{\partial \varphi }+\frac{1}{r}\left(2\sigma_{rr}-\sigma_{\vartheta \vartheta }-\sigma_{\varphi \varphi }+\sigma_{r\vartheta }\text{cot}\vartheta \right)=0, \end{array}$$
  (8) $$\begin{array}{r} \frac{\partial \sigma_{\vartheta r}}{\partial r}+\frac{1}{r}\frac{\partial \sigma_{\vartheta \vartheta }}{\partial \vartheta }+\frac{1}{r\text{sin}\vartheta }\frac{\partial \sigma_{\vartheta \varphi }}{\partial \varphi }+\frac{1}{r}\left[\left(\sigma_{\vartheta \vartheta }-\sigma_{\varphi \varphi }\right)\text{cot}\vartheta +3\sigma_{r\vartheta }\right]=0, \end{array}$$
  (9) $$\begin{array}{r} \frac{\partial \sigma_{\varphi r}}{\partial r}+\frac{1}{r}\frac{\partial \sigma_{\varphi \vartheta }}{\partial \vartheta }+\frac{1}{r\text{sin}\vartheta }\frac{\partial \sigma_{\varphi \varphi }}{\partial \varphi }+\frac{1}{r}\left(3\sigma_{\varphi r}+2\sigma_{\varphi \vartheta }\text{cot}\vartheta \right)=0. \end{array}$$
- Kinematic relation:
  (10) $$\begin{array}{l} 0\leq r<\infty :\;\varepsilon =\nabla^{\text{s}}\xi , \end{array}$$
  where ∇^s^ represents the symmetric gradient operator. Evaluation of Equation (10) in spherical coordinates yields (Salençon, 2001)
  (11) $$\begin{array}{l} \varepsilon =\left(\begin{array}{ll} \frac{\partial \xi_{r}}{\partial r}\frac{1}{2}\left(\frac{1}{r}\frac{\partial \xi_{r}}{\partial \vartheta }+\frac{\partial \xi_{\vartheta }}{\partial r}-\frac{\xi_{\vartheta }}{r}\right) & \;\frac{1}{2}\left(\frac{1}{r\text{sin}\vartheta }\frac{\partial \xi_{r}}{\partial \varphi }+\frac{\partial \xi_{\varphi }}{\partial r}-\frac{\xi_{\varphi }}{r}\right)\\ \;\frac{1}{r}\frac{\partial \xi_{\vartheta }}{\partial \vartheta }+\frac{\xi_{r}}{r} & \frac{1}{2}\left(\frac{1}{r}\frac{\partial \xi_{\varphi }}{\partial \vartheta }+\frac{1}{r\text{sin}\vartheta }\frac{\partial \xi_{\vartheta }}{\partial \varphi }-\frac{\text{cot}\vartheta }{r}\xi_{\varphi }\right)\\ \text{symm}. & \;\frac{1}{r\text{sin}\vartheta }\frac{\partial \xi_{\varphi }}{\partial \varphi }+\frac{\xi_{\vartheta }}{r}\text{cot}\vartheta +\frac{\xi_{r}}{r} \end{array}\right). \end{array}$$

The nature of the coated inclusion is reflected by stiffness properties being defined as functions of the radius measured from the center of that inclusion:

(12) $$\begin{array}{rc} r\leq r_{\text{gran}}: & \sigma \left(r\right)={\mathbb{C}}_{\text{gran}}:\varepsilon \left(r\right), \end{array}$$

(13) $$\begin{array}{rc} r_{\text{gran}}\leq r\leq r_{\text{bone}}: & \sigma \left(r\right)={\mathbb{C}}_{\text{bone}}:\varepsilon \left(r\right), \end{array}$$

(14) $$\begin{array}{rc} r\geq r_{\text{bone}}: & \sigma \left(r\right)={\mathbb{C}}_{\text{congl}}:\varepsilon \left(r\right), \end{array}$$

with *r*_gran_ and *r*_bone_ denoting the radii of the individual granule and of the outer surface of the bone coating; with **σ**(*r*) and **ε**(*r*) denoting the stresses and strains prevailing in the Hervé-Zaoui coated inclusion problem; with ℂ_gran_ as the isotropic stiffness tensor of the granule material, determined according to Equation (3); with ℂ_bone_ as the stiffness tensor of newly grown bone tissue; and with ℂ_congl_ as the stiffness of the overall macroporous scaffold-bone compound of hierarchical level III. Due to the random orientation of the morphological features on all considered observation scales (hierarchical levels I – III), the overall conglomerate stiffness is isotropic, hence it is defined as

(15) $$\begin{array}{l} {\mathbb{C}}_{\text{congl}}=3k_{\text{congl}}𝕂+2\mu_{\text{congl}}𝕁 \end{array}$$

with *k*_congl_ and μ_congl_ as the bulk and shear modulus of the scaffold-bone conglomerate on the macroscopic observation scale (hierarchical level III).

As regards the stiffness tensor of newly formed bone tissue, ℂ_bone_, we adapt the strategy described in Bertrand and Hellmich (2009) for the current purpose: We start with the ultrasound-based tissue stiffness reported by Ashman and van Buskirk (1987), amounting to

(16) $$\begin{array}{l} \begin{array}{c} {\mathbb{C}}_{\text{bone}}^{\text{us}}=\left(\begin{array}{cccccc} C_{\text{bone},\text{1111}} & C_{\text{bone},\text{1122}} & C_{\text{bone},\text{1133}} & \sqrt{2}C_{\text{bone},\text{1123}} & \sqrt{2}C_{\text{bone},\text{1113}} & \sqrt{2}C_{\text{bone},\text{1112}}\\ C_{\text{bone},\text{2211}} & C_{\text{bone},\text{2222}} & C_{\text{bone},\text{2233}} & \sqrt{2}C_{\text{bone},\text{2223}} & \sqrt{2}C_{\text{bone},\text{2213}} & \sqrt{2}C_{\text{bone},\text{2212}}\\ C_{\text{bone},\text{3311}} & C_{\text{bone},\text{3322}} & C_{\text{bone},\text{3333}} & \sqrt{2}C_{\text{bone},\text{3323}} & \sqrt{2}C_{\text{bone},\text{3313}} & \sqrt{2}C_{\text{bone},\text{3312}}\\ \sqrt{2}C_{\text{bone},\text{2311}} & \sqrt{2}C_{\text{bone},\text{2322}} & \sqrt{2}C_{\text{bone},\text{2333}} & 2C_{\text{bone},\text{2323}} & 2C_{\text{bone},\text{2313}} & 2C_{\text{bone},\text{2312}}\\ \sqrt{2}C_{\text{bone},\text{1311}} & \sqrt{2}C_{\text{bone},\text{1322}} & \sqrt{2}C_{\text{bone},\text{1333}} & 2C_{\text{bone},\text{1323}} & 2C_{\text{bone},\text{1313}} & 2C_{\text{bone},\text{1312}}\\ \sqrt{2}C_{\text{bone},\text{1211}} & \sqrt{2}C_{\text{bone},\text{1222}} & \sqrt{2}C_{\text{bone},\text{1233}} & 2C_{\text{bone},\text{1223}} & 2C_{\text{bone},\text{1213}} & 2C_{\text{bone},\text{1212}} \end{array}\right)=\left(\begin{array}{cccccc} 15.90 & 8.33 & 9.79 & 0 & 0 & 0\\ 8.33 & 18.8 & 9.79 & 0 & 0 & 0\\ 9.79 & 9.79 & 27.10 & 0 & 0 & 0\\ 0 & 0 & 0 & 9.26 & 0 & 0\\ 0 & 0 & 0 & 0 & 8.24 & 0\\ 0 & 0 & 0 & 0 & 0 & 7.62 \end{array}\right)\text{GPa}, \end{array} \end{array}$$

where the directions 1, 2, and 3 refer to the radial, circumferential, and axial directions of the orthotropic bone tissue material. The axial direction is aligned with the collagen fibril orientation, and during bone regeneration, the latter follows given morphological features occurring under normal physiological conditions. Since the spherical granular features deviate from the aforementioned physiological situation, it is very probable that the collagen fibrils are oriented along the tangent planes to the granule spheres, while not having any preferred orientation within these planes. As a first-order approximation of this situation, we let the stiffness tensor given by Equation (16) rotate first around axis 3 and average over all corresponding results (this leads to a transversely isotropic stiffness tensor with the isotropic plane coinciding with the 1–2 plane), and we then let the latter stiffness tensor rotate about an axis orthogonal to axis 3, which in turn leads to yet another transversely isotropic stiffness tensor with the plane of isotropy now including the axis 3. With respect to a spherical coordinate system attached to the granule, see Figure 2, the isotropic plane of the newly grown bone tissue coincides with the **e**_ϑ_-**e**_φ_-plane, so that ℂ_bone_ can be given as

(17) $$\begin{array}{l} \begin{array}{c} C_{\text{bone}}=\left(\begin{array}{cccccc} C_{\text{bone},rrrr} & C_{\text{bone},rr\vartheta \vartheta } & C_{\text{bone},rr\varphi \varphi } & 0 & 0 & 0\\ C_{\text{bone},rr\vartheta \vartheta } & C_{\text{bone},\vartheta \vartheta \vartheta \vartheta } & C_{\text{bone},\vartheta \vartheta \varphi \varphi } & 0 & 0 & 0\\ C_{\text{bone},rr\varphi \varphi } & C_{\text{bone},\vartheta \vartheta \varphi \varphi } & C_{\text{bone},\varphi \varphi \varphi \varphi } & 0 & 0 & 0\\ 0 & 0 & 0 & 2C_{\text{bone},\vartheta \varphi \vartheta \varphi } & 0 & 0\\ 0 & 0 & 0 & 0 & 2C_{\text{bone},r\varphi r\varphi } & 0\\ 0 & 0 & 0 & 0 & 0 & 2C_{\text{bone},r\vartheta r\vartheta } \end{array}\right)=\left(\begin{array}{cccccc} 15.90 & 9.00 & 9.00 & 0 & 0 & 0\\ 9.00 & 21.74 & 10.70 & 0 & 0 & 0\\ 9.00 & 10.70 & 21.74 & 0 & 0 & 0\\ 0 & 0 & 0 & 11.04 & 0 & 0\\ 0 & 0 & 0 & 0 & 7.93 & 0\\ 0 & 0 & 0 & 0 & 0 & 7.93 \end{array}\right)\text{GPa}. \end{array} \end{array}$$

Given the isotropic nature of the overall scaffold-bone compound material, our interest in auxiliary homogenous strain fields **E**_0_ prescribed at the infinite boundary of the Hervé-Zaoui matrix according to Equation (6) can be restricted to purely volumetric and purely deviatoric cases; as will become fully evident during the bulk and shear modulus homogenization described in Section 2.5.

For the volumetric load case, the boundary condition defined by Equation (6) is restricted to

(18) $$\begin{array}{l} r\to \infty :\;{\text{E}}_{0}={\text{E}}_{\text{vol},0}=\frac{E_{\text{vol},0}}{3}\text{I}\;\Rightarrow \;\xi_{0}=\xi_{\text{vol},0}=r\frac{E_{\text{vol},0}}{3}{\text{e}}_{r} \end{array}$$

where **I** is the second-order unit tensor, and **e**_*r*_ is the base vector in radial direction, see Figure 2. Spherical symmetry of the remote loading conditions according to Equation (18) implies spherical symmetry of the resulting displacement field,

(19) $$\begin{array}{l} \xi =\xi \left(r\right)=\xi_{r}\left(r\right){\text{e}}_{r}, \end{array}$$

with the corresponding spherically symmetric strain field reading as

(20) $$\begin{array}{l} \varepsilon =\frac{\partial \xi_{r}\left(r\right)}{\partial r}{\text{e}}_{r}⊗{\text{e}}_{r}+\frac{\partial \xi_{r}\left(r\right)}{\partial \vartheta }{\text{e}}_{\vartheta }⊗{\text{e}}_{\vartheta }+\frac{\partial \xi_{r}\left(r\right)}{\partial \varphi }{\text{e}}_{\varphi }⊗{\text{e}}_{\varphi }. \end{array}$$

Insertion of this strain field into the elastic laws given by Equations (12) – (14), and of the corresponding result into the equilibrium condition given by Equations (6) – (9) yields

(21) $$\begin{array}{l} \frac{{\text{d}}^{2}\xi_{r}}{\text{d}r^{2}}+\frac{2}{r}\frac{\text{d}\xi_{r}}{\text{d}r}-\frac{2\left(C_{i,\vartheta \vartheta \vartheta \vartheta }+C_{i,\vartheta \vartheta \varphi \varphi }-C_{i,rr\vartheta \vartheta }\right)}{C_{i,rrrr}}\frac{\xi_{r}}{r^{2}}=0. \end{array}$$

When introducing parameter *n*_*i*_,

(22) $$\begin{array}{l} n_{i}=\sqrt{\frac{1}{4}+\frac{2\left(C_{i,\vartheta \vartheta \vartheta \vartheta }+C_{i,\vartheta \vartheta \varphi \varphi }-C_{i,rr\vartheta \vartheta }\right)}{C_{i,rrrr}}}, \end{array}$$

the general solution of the ordinary differential equation (21) reads as

(23) $$\begin{array}{l} \xi_{i,r}\left(r\right)=\Gamma_{i,1}^{k}r^{-1/2+n_{i}}+\Gamma_{i,2}^{k}r^{-1/2-n_{i}}. \end{array}$$

For the definitions of material parameters $\Gamma_{i,j}^{k}$ (*j* = 1, 2), see the Supplementary Material. Evaluating Equation (22) for an isotropic phase, where $C_{i,rrrr}^{\text{iso}}=C_{i,\vartheta \vartheta \vartheta \vartheta }^{\text{iso}}=k_{i}+\frac{4}{3}\mu_{i}$, and $C_{i,\vartheta \vartheta \varphi \varphi }^{\text{iso}}=C_{i,rr\vartheta \vartheta }^{\text{iso}}=k_{i}-\frac{2}{3}\mu_{i}$, one can easily see that *n*_*i*_ = 3/2; this is the case for *i* = gran, scaff. For the bone tissue stiffness according the Equation (17), *n*_bone_ amounts to 1.79. Inserting the general solution of the displacement field, Equation (23), into the kinematic relation, Equation (11), yields the corresponding strain field, given by its components in spherical coordinates,

(24) $$\begin{array}{l} \varepsilon_{i,rr}\left(r\right)=\left(-\frac{1}{2}+n_{i}\right)\Gamma_{i,1}^{k}r^{-3/2+n_{i}}\\ \text{ +}\left(-\frac{1}{2}-n_{i}\right)\Gamma_{i,2}^{k}r^{-3/2-n_{i}}, \end{array}$$

(25) $$\begin{array}{r} \varepsilon_{i,\vartheta \vartheta }\left(r\right)=\Gamma_{i,1}^{k}r^{-3/2+n_{i}}+\Gamma_{i,2}^{k}r^{-3/2-n_{i}}, \end{array}$$

(26) $$\begin{array}{r} \varepsilon_{i,\varphi \varphi }\left(r\right)=\varepsilon_{i,\vartheta \vartheta }\left(r\right). \end{array}$$

The stress field resulting from hydrostatic deformations follows from insertion of Equations (24) – (26) into the constitutive relations, Equations (12) – (14), as

(27) $$\begin{array}{r} \sigma_{i,rr}\left(r\right)=r^{-3/2}\left(M_{i}\Gamma_{i,1}^{k}r^{n_{i}}+N_{i}\Gamma_{i,2}^{k}r^{-n_{i}}\right), \end{array}$$

(28) $$\begin{array}{r} \sigma_{i,\vartheta \vartheta }\left(r\right)=r^{-3/2}\left(O_{i}\Gamma_{i,1}^{k}r^{n_{i}}+P_{i}\Gamma_{i,2}^{k}r^{-n_{i}}\right), \end{array}$$

(29) $$\begin{array}{r} \sigma_{i,\varphi \varphi }\left(r\right)=\sigma_{i,\vartheta \vartheta }\left(r\right). \end{array}$$

For the definitions of material constants M_*i*_, N_*i*_, O_*i*_, and P_*i*_, see the Supplementary Material.

As regards the deviatoric loading, we prescribe a purely deviatoric (pure shear) deformation **E**_d, 0_ at the infinitely remote boundary of the domain depicted in Figure 2,

(30) $$\begin{array}{l} r\to \infty :{\text{E}}_{\text{d},0}=\gamma \left({\text{e}}_{x}⊗{\text{e}}_{x}-{\text{e}}_{y}⊗{\text{e}}_{y}\right), \end{array}$$

where **e**_*x*_ and **e**_*y*_ are the base vectors of a Cartesian coordinate system with its origin in the center of the granule, and symbol ⊗ represents the dyadic vector product. The related displacements at the infinitely remote boundary follow as

(31) $$\begin{array}{l} \xi_{\text{d},0}=\gamma (xe_{x}-ye_{y})\\ \;=\gamma [(r\sin^{2}\vartheta \;\cos2\varphi )e_{r}+(r\sin\vartheta \;\cos\vartheta \;\cos2\varphi )e_{\vartheta }\\ \;+(r\sin\vartheta \;\sin2\varphi )e_{\varphi }], \end{array}$$

with **e**_*r*_, **e**_ϑ_, and **e**_φ_ as the base vectors of a spherical coordinate system, also originating from the granule center. Furthermore, the displacement field across the whole domain reads as

(32) $$\begin{array}{cc} \begin{array}{l} \Xi =\Xi (r,\vartheta ,\varphi )\\ \;=[\xi_{r}(r)\sin^{2}\vartheta \cos2\varphi ]e_{r}+[\xi_{\vartheta }(r)\sin\vartheta \cos\vartheta \cos2\varphi ]e_{\vartheta }\\ \;+[\xi_{\varphi }(r)\sin\vartheta \sin2\varphi ]e_{\varphi }. \end{array} & \left(32\right) \end{array}$$

The displacement field is then inserted into Equation (11). The resulting strain field enters again the constitutive relations, Equations (12) – (14), and the obtained stress field gives access, via the equilibrium condition, Equations (6) – (9), to a set of ordinary differential equations, which can be solved for the components of the displacement field, see the Supplementary Material. The general solutions of the set of differential equations read

(33) $$\begin{array}{r} \xi_{i,r}\left(r\right)=\overset{4}{\underset{j=1}{\sum }}\Gamma_{i,j}^{\mu }r^{-\frac{1}{2}-\alpha_{i,j}}, \end{array}$$

(34) $$\begin{array}{r} \xi_{i,\vartheta }\left(r\right)=\overset{4}{\underset{j=1}{\sum }}\beta \left(\alpha_{i,j}\right)\Gamma_{i,j}^{\mu }r^{-\frac{1}{2}-\alpha_{i,j}}, \end{array}$$

(35) $$\begin{array}{r} \xi_{i,\varphi }\left(r\right)=-\xi_{i,\vartheta }\left(r\right), \end{array}$$

with β(α_*i, j*_) defined as

(36) $$\begin{array}{l} \beta \left(\alpha_{i,j}\right)=-\frac{P_{i,11}\left(\alpha_{i,j}\right)}{P_{i,12}\left(\alpha_{i,j}\right)}. \end{array}$$

The underlying parameters, i.e., α_*i, j*_, *P*_*i*, 11_(α_*i, j*_), and *P*_*i*, 12_(α_*i, j*_), are defined in the Supplementary Material. Note that, in line with Bertrand and Hellmich (2009), Equation (33) – (35) are subsequently employed for quantification of the displacement field in the anisotropic constituents only (i.e., solely in the bone phase). For the isotropic constituents within the RVE defined on hierarchical level III, *i*_iso_ = gran, congl, the displacement field solutions originally given by Hervé and Zaoui (1993) are employed:

(37) $$\begin{array}{r} \xi_{i_{\text{iso}},r}\left(r\right)=\Gamma_{i_{\text{iso}},1}^{\mu }r-6\frac{\nu_{i_{\text{iso}}}}{1-2\nu_{i_{\text{iso}}}}\Gamma_{i_{\text{iso}},2}^{\mu }r^{3}+3\frac{\Gamma_{i_{\text{iso}},3}^{\mu }}{r^{4}}\\ +\frac{5-4\nu_{i_{\text{iso}}}}{1-2\nu_{i_{\text{iso}}}}\frac{\Gamma_{i_{\text{iso}},4}^{\mu }}{r^{2}}, \end{array}$$

(38) $$\begin{array}{r} \xi_{i_{\text{iso}},\vartheta }\left(r\right)=\Gamma_{i_{\text{iso}},1}^{\mu }r-\frac{7-4\nu_{i_{\text{iso}}}}{1-2\nu_{i_{\text{iso}}}}\Gamma_{i_{\text{iso}},2}^{\mu }r^{3}-2\frac{\Gamma_{i_{\text{iso}},3}^{\mu }}{r^{4}}+2\frac{\Gamma_{i_{\text{iso}},4}^{\mu }}{r^{2}}, \end{array}$$

(39) $$\begin{array}{r} \xi_{i_{\text{iso}},\varphi }\left(r\right)=-\xi_{i_{\text{iso}},\vartheta }\left(r\right). \end{array}$$

For determination of parameters $\Gamma_{i,j}^{\mu }$, see the Supplementary Material.

For estimating strain states in the macropores, the standard Eshelby matrix-inclusion problem (Eshelby, 1957) is considered, where the infinite matrix is subjected to the *same* strains as those in the Hervé-Zaoui problem. Under these conditions, the strains in a spherical inclusion with the stiffness properties of the macropores, ℂ_macroϕ_, which is embedded in a matrix with the stiffness ℂ_congl_ of the overall scaffold-bone conglomerate material, read as

(40) $$\begin{array}{l} \varepsilon_{\text{macro}\phi }^{\text{congl}}={\left\{𝕀+{𝕊}_{\text{sph}}^{\text{congl}}:\left[{\left({\mathbb{C}}_{\text{congl}}\right)}^{-1}:{\mathbb{C}}_{\text{macro}\phi }-I\right]\right\}}^{-1}:{\text{E}}_{\text{0}}, \end{array}$$

with ${𝕊}_{\text{sph}}^{\text{congl}}$ as the Eshelby tensor of a spherical inclusion embedded in a matrix with stiffness ${\mathbb{C}}_{\text{sph}}^{\text{congl}}$, see the Supplementary Material for more details. Notably, the macropores are, as micro- and mesopores, considered to be drained, i.e., ℂ_macroϕ_ = 0. The phase stresses corresponding to the phase strains given through Equation (40) follow from the elastic law of the macropores, reading as

(41) $$\begin{array}{l} \sigma_{\text{macro}\phi }^{\text{congl}}={\mathbb{C}}_{\text{macro}\phi }:\varepsilon_{\text{macro}\phi }^{\text{congl}}. \end{array}$$

### 2.5. Bulk and shear stiffness homogenization at hierarchical level iii: elasticity of macroporous granule-bone conglomerate

Homogenization of bulk and shear moduli of the overall conglomerate will be performed on the basis of (i) the microdisplacement, microstress, and microstrain fields in the granule, bone, and macropore phases as given through Equations (23) – (29), (33) – (39), as well as (40) and (41), of (ii) the stress and strain average rules applied to the RVE III of Figure 2, and of (iii) the definition of the bulk and the shear modulus in the terms of macroscopic stress and strain measures. The aforementioned stress and strain average rules, enforcing equilibrium and kinematic compatibility within the RVE III, read as

(42) $$\begin{array}{l} \Sigma_{\text{congl}}=\frac{1}{V_{\text{RVE III}}}\underset{V_{\text{RVE III}}}{\int }\sigma \left(\text{x}\right)\text{d}V, \end{array}$$

and

(43) $$\begin{array}{l} {\text{E}}_{\text{congl}}=\frac{1}{V_{\text{RVE III}}}\underset{V_{\text{RVE III}}}{\int }\varepsilon \left(\text{x}\right)\text{d}V, \end{array}$$

with the location vector **x** labeling points inside the RVE III. By definition, the bulk modulus describes the volume change of a material volume if it is subjected to hydrostatic pressure. Accordingly, the bulk modulus of the scaffold-bone compound material with a stiffness according to Equation (15) is defined as

(44) $$\begin{array}{l} k_{\text{congl}}=\frac{\Sigma_{\text{congl},\text{m}}}{E_{\text{congl},\text{vol}}}, \end{array}$$

where Σ_congl, m_ is the mean macroscopic stress of the scaffold material, Σ_congl, m_ = tr**Σ**_congl_/3, and *E*_congl, vol_ is the macroscopic volumetric strain of the scaffold material, *E*_congl, vol_ = tr**E**_congl_; “tr” denoting the trace operator. Dividing the RVE III into the material phases introduced in Section 2.1 and Figure 1, the mean conglomerate stresses and the volumetric conglomerate strains can be expressed by means of the stress and strain average rules defined by Equations (42) and (43), yielding

(45) $$\begin{array}{l} \sum_{\text{congl},\text{m}}=f_{\text{gran}}^{\text{congl}}{\langle \sigma_{\text{m}}(x)\rangle }_{\text{gran}}^{\text{congl}}+f_{\text{bone}}^{\text{congl}}{\langle \sigma_{\text{m}}(x)\rangle }_{\text{bone}}^{\text{congl}}\\ \;+\phi_{\text{macro}}^{\text{congl}}{\langle \sigma_{\text{m}}(x)\rangle }_{\text{macro}\phi }^{\text{congl}}, \end{array}$$

(46) $$\begin{array}{l} E_{\text{congl},\text{vol}}=f_{\text{gran}}^{\text{congl}}{\langle \varepsilon_{\text{vol}}(x)\rangle }_{\text{gran}}^{\text{congl}}+f_{\text{bone}}^{\text{congl}}{\langle \varepsilon_{\text{vol}}(x)\rangle }_{\text{bone}}^{\text{congl}}\\ \;+\phi_{\text{macro}}^{\text{congl}}{\langle \varepsilon_{\text{vol}}(x)\rangle }_{\text{macro}\phi }^{\text{congl}}. \end{array}$$

where $f_{\text{gran}}^{\text{congl}}$, $f_{\text{bone}}^{\text{congl}}$, and $\phi_{\text{macro}}^{\text{congl}}$ are the volume fractions of the granules, of the newly formed bone tissue, and of the macropores, respectively; ${\langle \sigma_{\text{m}}\left(x\right)\rangle }_{\text{gran}}^{\text{congl}}$, ${\langle \sigma_{\text{m}}\left(x\right)\rangle }_{\text{bone}}^{\text{congl}}$, and ${\langle \sigma_{\text{m}}\left(x\right)\rangle }_{\text{macro}\phi }^{\text{congl}}$ are the volume averages of the mean microscopic stresses in the granules, in the newly formed bone tissue, and in the macropores, respectively; and ${\langle \varepsilon_{\text{vol}}\left(x\right)\rangle }_{\text{gran}}^{\text{congl}}$, ${\langle \varepsilon_{\text{vol}}\left(x\right)\rangle }_{\text{bone}}^{\text{congl}}$, and ${\langle \varepsilon_{\text{vol}}\left(x\right)\rangle }_{\text{macro}\phi }^{\text{congl}}$ are the volume averages of the volumetric microscopic strains in the granules, in the newly formed bone tissue, and in the macropores, respectively. The phase strain averages occurring in Equation (46) are then approximated by the microstrain fields obtained from the matrix-(coated or non-coated) inclusion problems introduced in Section 2.4. I.e., Equation (40) is evaluated for **E**_0_ = **E**_vol, 0_, so as to arrive at

(47) $$\begin{array}{l} {\langle \varepsilon_{\text{vol}}(x)\rangle }_{\text{macro}\phi }^{\text{congl}}\\ \;=\text{tr }\left[{\left\{𝕀+{𝕊}_{\text{sph}}^{\text{congl}}:\left[{\left({\mathbb{C}}_{\text{congl}}\right)}^{-1}:{\mathbb{C}}_{\text{macro}\phi }-𝕀\right]\right\}}^{-1}:E_{\text{vol},0}\right]\\ \;=\frac{3k_{\text{congl}}+4\mu_{\text{congl}}}{3k_{\text{macro}\phi }+4\mu_{\text{macro}\phi }}E_{\text{vol},0}, \end{array}$$

and the corresponding mean stress follows as

(48) $$\begin{array}{l} {\langle \sigma_{\text{m}}(x)\rangle }_{\text{macro}\phi }^{\text{congl}}=k_{\text{macro}\phi }{\langle \varepsilon_{\text{vol}}(x)\rangle }_{\text{macro}\phi }^{\text{congl}}, \end{array}$$

with *k*_macroϕ_ and μ_macroϕ_ as the bulk and shear moduli of the macropore phase. The average volumetric phase strains in the granule and bone tissue phases, respectively, are obtained from averaging over strains occurring in the granules, and of the bone tissue coating of the Hervé-Zaoui problem of Figure 2, according to

(49) $$\begin{array}{l} {\langle \varepsilon_{\text{vol}}(x)\rangle }_{i}^{\text{congl}}=\text{tr}[\frac{1}{V_{i}}\int_{r_{i,\text{in}}}^{r_{i,\text{out}}}\int_{0}^{\pi }\int_{0}^{2\pi }r^{2}\varepsilon (r)\sin\vartheta \text{ d}\vartheta \text{ d}\varphi \text{ d}r]. \end{array}$$

For the evaluation of Equation (49), it is mandatory that all strain tensors are expressed in the *same* (Cartesian) coordinate system; also in Equation (49), *r*_*i*, in_ and *r*_*i*, out_ denote the inner and outer radii of the respective layer: e.g., *i* = bone gives *r*_bone, in_ = *r*_gran_ and *r*_bone, out_ = *r*_bone_, see Figure 2. Analogously, the phase volume averages of the mean stresses in the granule and bone phases follow from the stress field components given by Equations (27) – (29) as

(50) $$\begin{array}{l} {\langle \sigma_{\text{m}}(x)\rangle }_{i}^{\text{congl}}=\frac{1}{3}\text{tr}[\frac{1}{V_{i}}\int_{r_{i,\text{in}}}^{r_{i,\text{out}}}\int_{0}^{\pi }\int_{0}^{2\pi }r^{2}\sigma (r)\sin\vartheta \text{ d}\vartheta \text{ d}\varphi \text{ d}r]. \end{array}$$

The mathematical expressions resulting from evaluating Equations (49) and (50) for the granule and the bone phases are given in the Supplementary Material. Finally, inserting Equations (47) – (50) into Equations (45) and (46), and of the resulting expression into Equation (44), yields an implicit equation for calculation of *k*_congl_.

The macrosopic shear modulus of the scaffold material is governed by another classical relation of continuum mechanics, namely

(51) $$\begin{array}{l} \mu_{\text{congl}}=\frac{\sum_{\text{congl},\text{d},ij}}{2E_{\text{congl},\text{d},ij}}, \end{array}$$

hence μ_congl_ follows from arbitrary components (with *i* ≠ *j*) of the deviatoric part of the macroscopic stress tensor, **Σ**_congl,d_, defined through **Σ**_congl,d_ = **Σ**_congl_ − **I**Σ_congl, m_, and the deviatoric part of the macroscopic strain tensor, **E**_congl,d_, defined through **E**_congl,d_ = **E**_congl_ − **I***E*_congl, m_. Taking into account that the scaffold material is a composite as defined in Figure 2, **Σ**_congl,d_ and **E**_congl,d_ follow from volume averaging as

(52) $$\begin{array}{l} \sum_{\text{congl},\text{d}}=f_{\text{gran}}^{\text{congl}}{\langle \sigma_{\text{d}}(x)\rangle }_{\text{gran}}^{\text{congl}}+f_{\text{bone}}^{\text{congl}}{\langle \sigma_{\text{d}}(x)\rangle }_{\text{bone}}^{\text{congl}}\\ \;+\phi_{\text{macro}}^{\text{congl}}{\langle \sigma_{\text{d}}(x)\rangle }_{\text{macro}\phi }^{\text{congl}}, \end{array}$$

(53) $$\begin{array}{l} E_{\text{congl},\text{d}}=f_{\text{gran}}^{\text{congl}}{\langle \varepsilon_{\text{d}}(x)\rangle }_{\text{gran}}^{\text{congl}}+f_{\text{bone}}^{\text{congl}}{\langle \varepsilon_{\text{d}}(x)\rangle }_{\text{bone}}^{\text{congl}}\\ \;+\phi_{\text{macro}}^{\text{congl}}{\langle \varepsilon_{\text{d}}(x)\rangle }_{\text{macro}\phi }^{\text{congl}}, \end{array}$$

with ${\langle \sigma_{\text{d}}\left(x\right)\rangle }_{\text{gran}}^{\text{congl}}$, ${\langle \sigma_{\text{d}}\left(x\right)\rangle }_{\text{bone}}^{\text{congl}}$, and ${\langle \sigma_{\text{d}}\left(x\right)\rangle }_{\text{macro}\phi }^{\text{congl}}$ as the volume averages of the deviatoric stress tensors across the granular material, bone, and macropore phases, and with ${\langle \varepsilon_{\text{d}}\left(x\right)\rangle }_{\text{gran}}^{\text{congl}}$, ${\langle \varepsilon_{\text{d}}\left(x\right)\rangle }_{\text{bone}}^{\text{congl}}$, and ${\langle \varepsilon_{\text{d}}\left(x\right)\rangle }_{\text{macro}\phi }^{\text{congl}}$ as the volume averages of the deviatoric strain tensors across the granular material, bone, and macropore phases. The strain tensor components, obtained through insertion of Equations (33) – (35) and Equations (37) – (39), respectively, into Equation (10), are then rotated to a Cartesian base frame, and averaged over the volumes of the individual phases *i*,

(54) $$\begin{array}{l} {\langle \varepsilon (\text{x})\rangle }_{i}=\frac{1}{V_{i}}\int_{r_{i,\text{in}}}^{r_{i,\text{out}}}\int_{0}^{\pi }\int_{0}^{2\pi }r^{2}\varepsilon (r)\sin\vartheta \text{ d}\vartheta \text{ d}\varphi \text{ d}r. \end{array}$$

Analogously, the volume average of stress tensors across one individual phase, whose components follow from insertion of the phase strain tensors into the constitutive relations, Equations (12) – (14),

(55) $$\begin{array}{l} {\langle \sigma \left(x\right)\rangle }_{i}=\frac{1}{V_{i}}\overset{r_{i,\text{out}}}{\underset{r_{i,\text{in}}}{\int }}\;\overset{\pi }{\underset{0}{\int }}\;\overset{2\pi }{\underset{0}{\int }}\;r^{2}\sigma (x)\sin\;\vartheta \text{ d}\vartheta \text{ d}\varphi \text{ d}r. \end{array}$$

Given that the here applied loading is purely deviatoric, the deviatoric strain and stress tensors are equal to the respective full tensors, that is ${\langle \varepsilon_{\text{d}}(x)\rangle }_{i}={\langle \varepsilon (x)\rangle }_{i}\text{ and }{\langle \sigma_{\text{d}}(x)\rangle }_{i}={\langle \sigma (x)\rangle }_{i}.$ The resulting expressions for ${\langle \varepsilon_{\text{d}}\left(x\right)\rangle }_{\text{gran}}^{\text{congl}}$, ${\langle \varepsilon_{\text{d}}\left(x\right)\rangle }_{\text{bone}}^{\text{congl}}$, ${\langle \sigma_{\text{d}}\left(x\right)\rangle }_{\text{gran}}^{\text{congl}}$, and ${\langle \sigma_{\text{d}}\left(x\right)\rangle }_{\text{bone}}^{\text{congl}}$, are given in the Supplementary Material. The average deviatoric part of the strain tensor across the macropore phase follows again from the matrix-inclusion-type approach of Equation (40),

(56) $$\begin{array}{l} {\langle \varepsilon \left(x\right)\rangle }_{\text{macro}\phi }^{\text{congl}}={\left\{𝕀+{𝕊}_{\text{sph}}^{\text{congl}}:\left[{\left({\mathbb{C}}_{\text{congl}}\right)}^{-1}:{\mathbb{C}}_{\text{macro}\phi }-I\right]\right\}}^{-1}:E_{\text{d},0}. \end{array}$$

Due to the purely deviatoric loading, ${\langle \varepsilon_{\text{d}}\left(x\right)\rangle }_{\text{macro}\phi }^{\text{congl}}$ equals ${\langle \varepsilon \left(x\right)\rangle }_{\text{macro}\phi }^{\text{congl}}$, reading, when further evaluating Equation (56), as

(57) $$\begin{array}{l} {\langle \varepsilon_{\text{d}}\left(x\right)\rangle }_{\text{macro}\phi }^{\text{congl}}=\left(5\mu_{\text{congl}}\left(3k_{\text{congl}}+4\mu_{\text{congl}}\right)\right)E_{\text{d},0}\\ \;\times [(\mu_{\text{congl}}\left(9k_{\text{congl}}+8\mu_{\text{congl}}\right)\\ \text{ + }{6\mu_{\text{macro}\phi }\left(k_{\text{congl}}+2\mu_{\text{congl}}\right)]}^{-1}. \end{array}$$

The corresponding average deviatoric stress tensor follows as

(58) $$\begin{array}{l} {\langle \sigma_{\text{d}}\left(x\right)\rangle }_{\text{macro}\phi }^{\text{congl}}=2\mu_{\text{macro}\phi }{\langle \varepsilon_{\text{d}}\left(x\right)\rangle }_{\text{macro}\phi }^{\text{congl}}. \end{array}$$

The macroscopic shear modulus of the scaffold material, μ_congl_, follows then from insertion of Equations (54), (55), (57), and (58) into Equations (52) and (53), and of the resulting expression into Equation (51). Analogously to the scaffold bulk modulus, μ_congl_ is a function of the scaffold stiffness. This implies that determination of μ_congl_ has to occur in conjunction with determination of *k*_congl_, and calls for an implicit scheme.

### 2.6. Animal studies and biomaterial experiments, revealing kinetics of bone ingrowth and scaffold resorption

**Figure 3:**
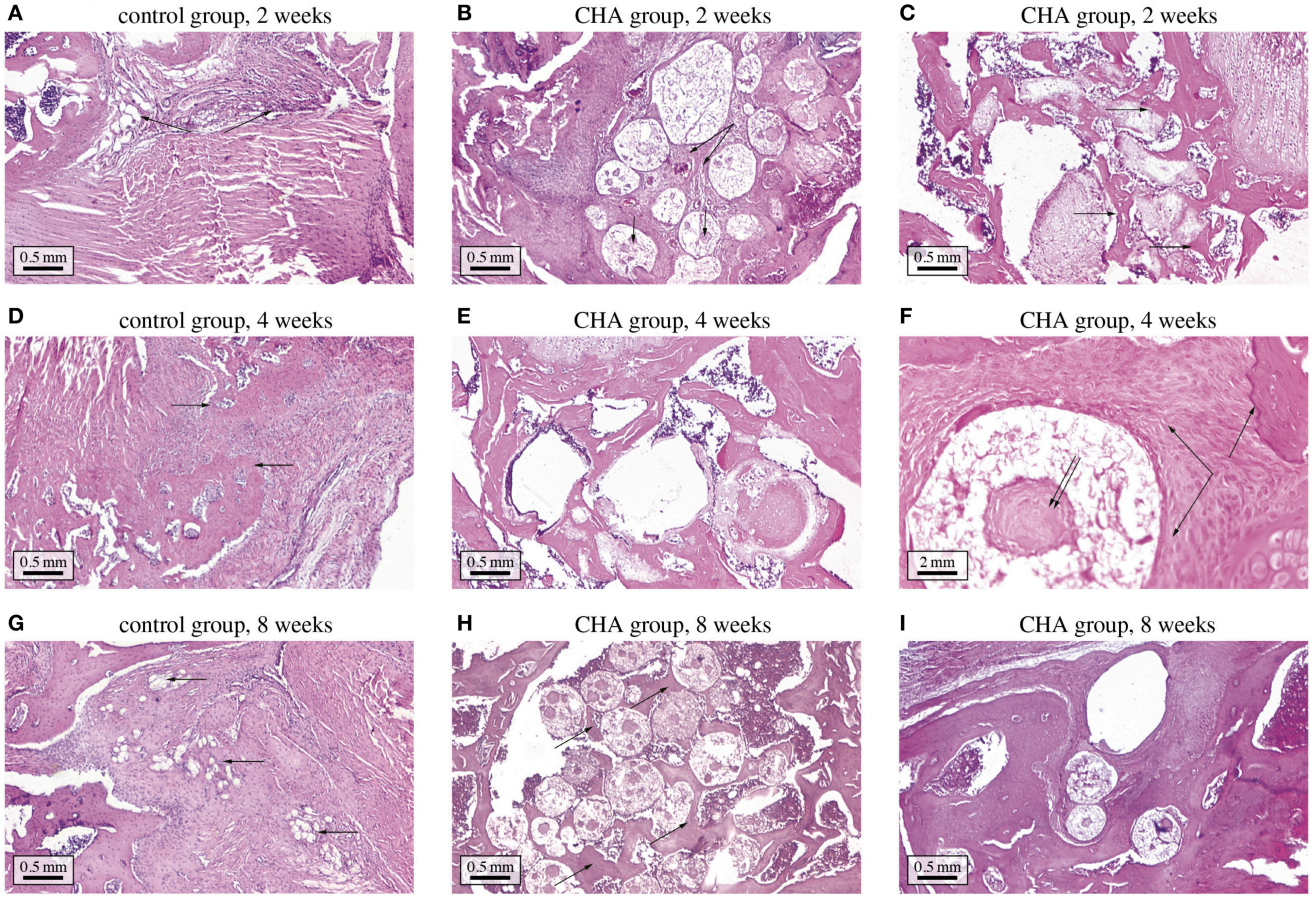
**Histological study of bone regeneration in bone defects optionally containing CHA granules. (A)** control group at 2 weeks after implantation—the bone defect is filled with connective tissue; **(B)** CHA group at 2 weeks after implantation—the granules are surrounded with connective tissue, in some granules osteoid formation can be discerned ; **(C)** CHA group at 2 weeks after implantation—near bone edges granules are surrounded with woven bone; **(D)** control group at 4 weeks after implantation—the bone defect is filled with connective tissue and woven bone; **(E)** CHA group at 4 weeks after implantation—the granules in the center of the defect are mostly surrounded with trabecular and woven bone; **(F)** CHA group at 4 weeks after implantation (higher magnification)—woven bone is forming around and inside CHA granule; **(G)** control group at 8 weeks after implantation—newly formed bone can be discerned at the edges of the bone defect; **(H)** CHA group at 8 weeks after implantation—trabecular bone has formed between CHA granules; **(I)** CHA group at 8 weeks after implantation—near bone edges granules are integrated in well-formed trabecular bone.

When applying the hydroxyapatite granule system *in vivo*, two out of the various phase volume fractions seen in Figure 2 and Section 2.1 significantly evolve over time: (i) the bone tissue volume fraction $f_{\text{bone}}^{\text{congl}}$, and (ii) the microporosity $\phi_{\text{micro}}^{\text{polyHA}}$. Corresponding evolutions can be estimated as follows:

As concerns bone ingrowth, we make use of insights gained from X-ray microtomography. Over a time span of roughly 10 weeks after implantation, the increase of the thickness of newly grown bone tissue on implanted biomaterials turned out to be approximately linear, at a rate of approximately *k*_growth_ = 4 μm/week (Cancedda et al., 2007). Given the near-spherical shape of the granules making up the biomaterial scaffold, the bone volume fraction evolution over time follows as

(59) $$\begin{array}{l} f_{\text{bone}}^{\text{congl}}=\left[\frac{{\left(r_{\text{gran}}+k_{\text{growth}}t\right)}^{3}}{r_{\text{gran}}^{3}}-1\right]f_{\text{gran}}^{\text{congl}}, \end{array}$$

where *t* is the time variable, *t* = 0 being the time instant of implantation, and *r*_gran_ the (average) radius of the granules. In order to define the bone deposition rate that is to be expected for the biomaterial studied in this paper, specific histological animal studies were carried out at the Central Scientific Research Institute of Dentistry and Maxillofacial Surgery (CRID), in Moscow, Russia. These studies were performed in conformity with the relevant institutional guidelines, which are in compliance with respective national laws and policies related to animal care, and which were approved by the Animal Ethics Committee of CRID. Thirty adult Wistar rats (12 weeks old, body weight 250 to 300 grams, and of both sexes) were divided into 2 groups. The rats were allowed free access to food and water *ad libitum* at all times and were maintained on a 12 h light/dark cycle (lights on from 8 a.m. and 8 p.m.). They were constantly exposed to a temperature of 23±1°C, and a relative humidity of 60±10 %. All rats were maintained and used in accordance with the guidelines of the Animal Ethics Committee of CRID For surgery, each rat was anesthetized by intraperitoneal injection of Zoletil 50 (Virbac S.A., France) with a dose of 0.2 mL/100 grams of body weight. For the femoral epiphysis model, an incision was made in the skin on the medial side of the thigh and the femoral quadriceps muscle was exposed. The muscle was sectioned longitudinally in its distal third and separated anterolaterally. After exposure of the distal end of the epiphysis of the right femur and peeling of the periosteum, a bone defect was created with a 3 mm hand-held surgical drill under irrigation of NaCl 0.9%. In the test group, carbonate hydroxyapatite (CHA) ceramic granules were inserted into the defect. In the control group, the bone defects were empty and healed under blood clots. Five animals of both groups were sacrificed at each 2, 4, and 8 weeks after implantation. For histological examination, the grafts with surrounding tissues were dissected and fixated in 10% buffered formalin for 24 h. Each graft was sectioned in ten pieces through its midline and embedded in paraffin. Serial 5 μm-sections were deparaffinized, hydrated and stained with hematoxylin and eosin. Photomicrographs of internal sections of each sample were taken by means of a light microscope (Leica DM LB, Germany) and photographed (Sony, Japan). Computer-assisted measurements of the histological parameters were obtained using an automated image analysis system Image-Pro Plus (Media Cybernetics, USA). For each graft at least 5 sections were examined, in each section at least 25 fields of vision were analyzed. The region of interest was taken at 100 × magnification and resolution of 2500 × 1200 pixels, see Figure 3. By measuring the percentage of newly formed bone (area) on the CHA test groups at 2, 4, and 8 weeks after implantation, revealing a bone bone ingrowth rate of *k*_growth_ = 7±3 μm/week, complying well with the aforementioned previous studies (Cancedda et al., 2007).

**Figure 4:**
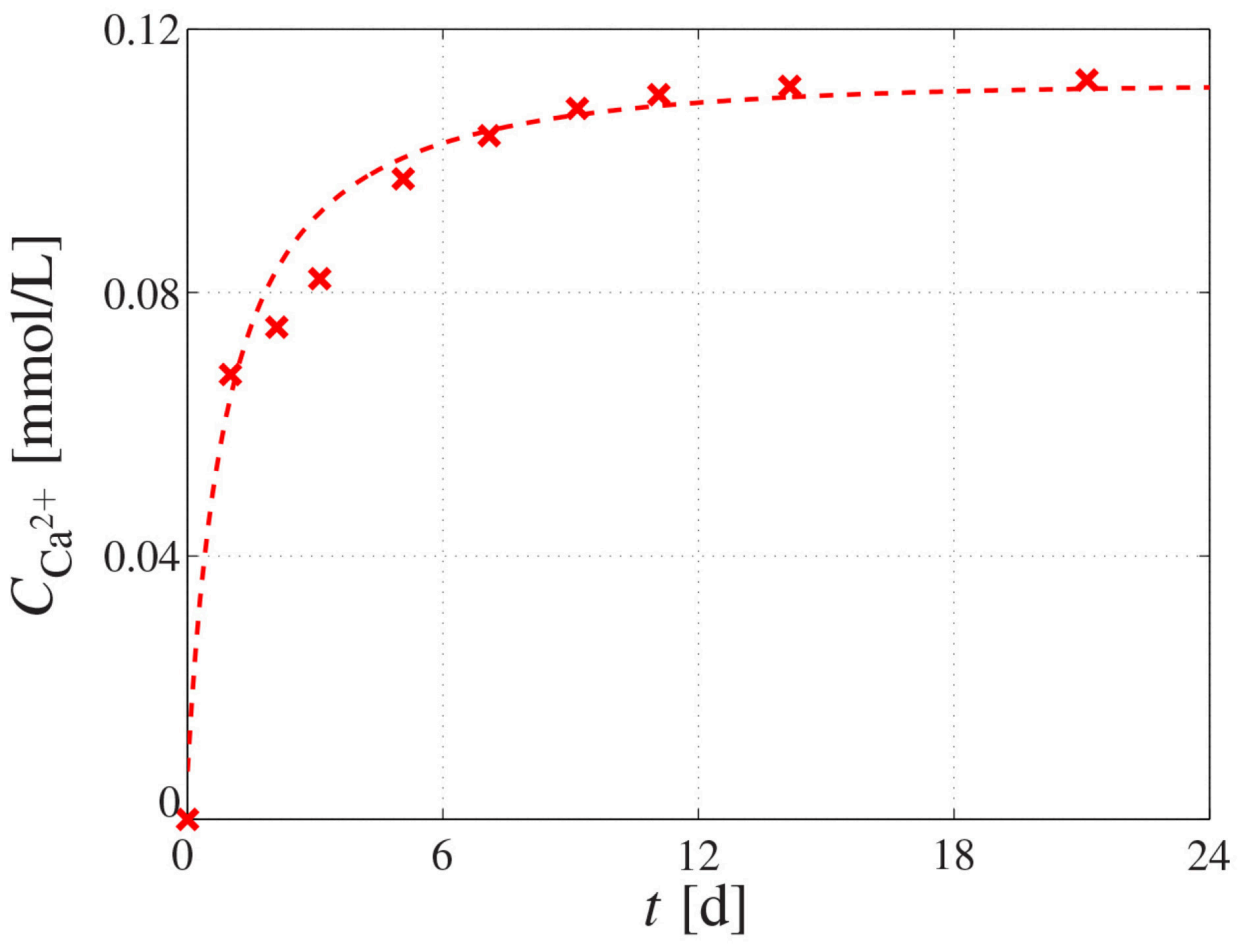
**Dissolution behavior of CHA granules**. Calcium concentration measured in the buffer solution by means of atomic emission spectrometry, reflecting the dissolution behavior of the immersed CHA granule; the cross-shaped markers represent the measured data, whereas the dashed graph represents the fitting of the experimental data by means of function $C_{{\text{Ca}}^{2+}}=\left(0.1073t^{2}+5.18t+0.3093\right)/\left(t^{2}+44.11t+42.61\right)$.

As concerns hydroxyapatite granule resorption, a recent μCT-based study on tricalcium phosphate biomaterials (Czenek et al., 2014) revealed that pseudo-physiological conditions favor micropore growth, while pores of larger sizes remain fairly unaffected. The corresponding temporal evolution of the microporosity during granule resorption thus reads as

(60) $$\begin{array}{l} \phi_{\text{micro}}^{\text{polyHA}}=\phi_{\text{micro},0}^{\text{polyHA}}+k_{\text{res}}t, \end{array}$$

with $\phi_{\text{micro},0}^{\text{polyHA}}$ as the microporosity before resorption sets in, and *k*_res_ as scaffold resorption rate. In order to find numerical values for the resorption rate, the solubility of CHA ceramic granules was studied in a TRIS-HCl buffer solution, exhibiting a *p*H-value of 7.4 (according to ISO 10993-14-2001) for 21 days at a constant liquid phase volume (thus representing a closed system). The desired *p*H-value was reached through adding 13.25 g of TRIS (Cat. No: 77-86-1, Sigma-Aldrich) and 125 ml of HCl (Cat. No: 7647-01-0, Aldrich-Aldrich). The solid-to-liquid ratio was 0.5 g/100 mL. The development of the calcium concentration over time in the liquid phase was measured using the atomic emission spectrometer Ultima 2 (Jobin-Yvon, France), see Figure 4. The corresponding dissolution rate of the scaffold material can be back-analyzed based on the chemical composition of the material, i.e., Ca_10_(PO_4_)_6_(OH)_2−2*x*_(CO_3_)_*x*_, with *x* = 0.05. Additionally, the gained experimental data was fitted by function $C_{{\text{Ca}}^{2+}}=\left(0.1073t^{2}+5.18t+0.3093\right)/\left(t^{2}+44.11t+42.61\right)$ (see the dashed graph in Figure 4), in order to get a continuous description of the dissolution progress. Considering the molecular masses of the hydroxyapatite crystals (which constitute the solid part of the granules), we back-calculated, through simple compositional rules, that the dissolution rate amounted to *k*_res_ ≈ 0.016 w^−1^ at the start of the dissolution process while, owing to the closed experimental system implying a changing buffer solution in the course of the dissolution process, the dissolution rate decreases thereafter, eventually converging to virtually zero.

## 3. Results and discussion

### 3.1. Microstructure-property relations: how micro/meso/macroporosity, crack density, and bone tissue volume fraction govern the overall scaffold-bone conglomerate stiffness

For elucidating the influence of the scaffold material composition on its stiffness, the latter was micromechanically estimated for the following parameter variations:

- The microporosity is varied between $\phi_{\text{micro}}^{\text{polyHA}}=\left[0.2,0.6\right]$, while $\phi_{\text{meso}}^{\text{gran}}=0.189$, ϵ = 10, $f_{\text{bone}}^{\text{congl}}=0$, and $\phi_{\text{macro}}^{\text{congl}}=\left[0.3,0.4,0.5\right]$;
- the mesoporosity is varied between $\phi_{\text{meso}}^{\text{gran}}=\left[0.1,0.3\right]$, while $\phi_{\text{micro}}^{\text{polyHA}}=0.445$, ϵ = 10, $f_{\text{bone}}^{\text{congl}}=0$, and $\phi_{\text{macro}}^{\text{congl}}=\left[0.3,0.4,0.5\right]$;
- the crack density parameter is varied between ϵ = [0, 100], while $\phi_{\text{micro}}^{\text{polyHA}}=0.445$, $\phi_{\text{meso}}^{\text{gran}}=0.189$, $f_{\text{bone}}^{\text{congl}}=0$, and $\phi_{\text{macro}}^{\text{congl}}=\left[0.3,0.4,0.5\right]$; and
- the bone tissue volume fraction is varied between $f_{\text{bone}}^{\text{congl}}=\left[0,0.5\right]$, while $\phi_{\text{micro}}^{\text{polyHA}}=0.445$, $\phi_{\text{meso}}^{\text{gran}}=0.189$, ϵ = 10, and $\phi_{\text{macro}}^{\text{congl}}=\left[0.3,0.4,0.5\right]$.

Furthermore, since stiffness of an isotropic material is often associated with the so-called Young's modulus, we present the computed bulk and shear modulus of the scaffold material in terms of the corresponding Young's modulus, through

(61) $$\begin{array}{l} E_{\text{congl}}=\frac{9k_{\text{congl}}\mu_{\text{congl}}}{3k_{\text{congl}}+\mu_{\text{congl}}}. \end{array}$$

**Figure 5:**
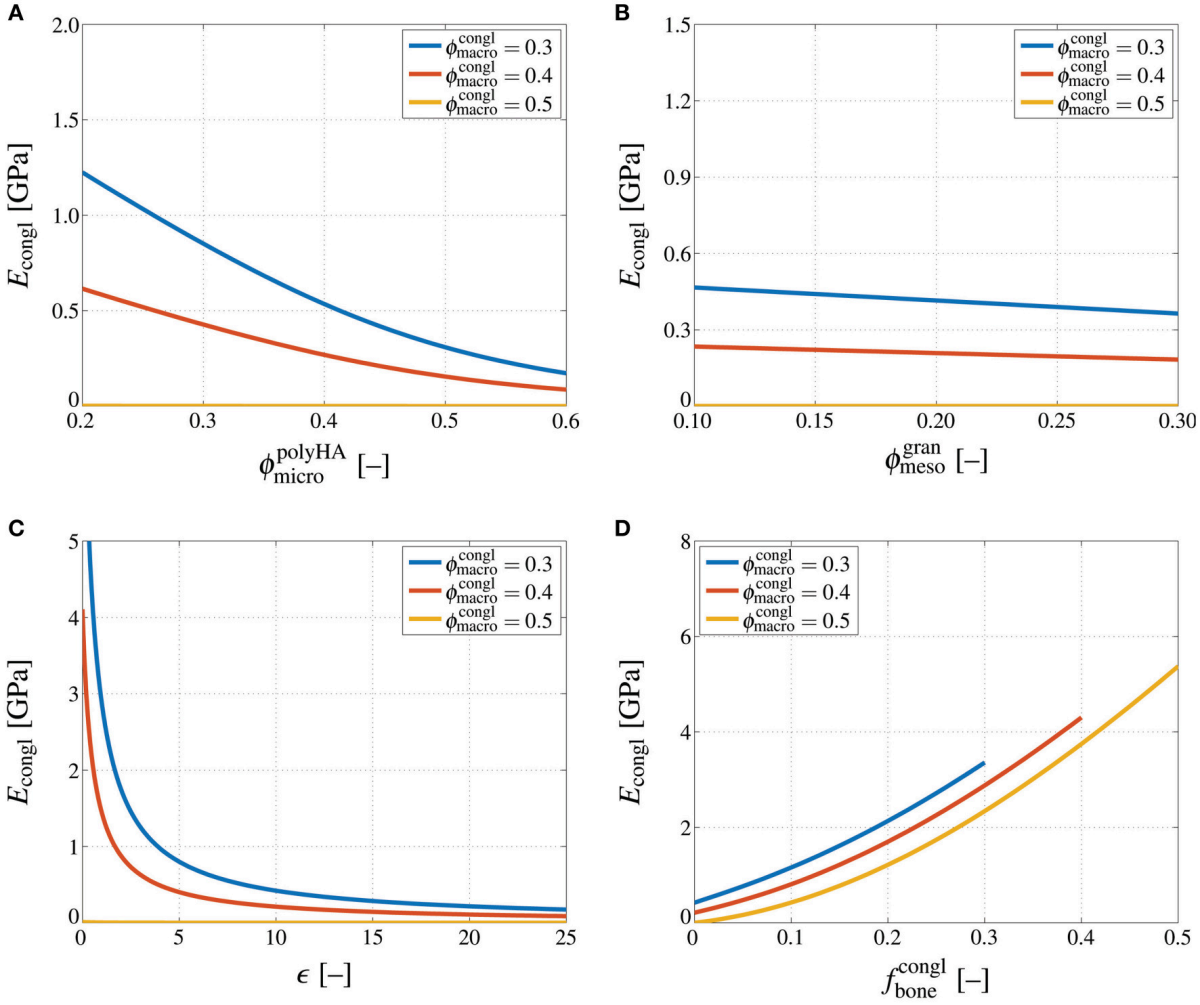
**Influences of porosities, crack density, and bone tissue volume fraction on the conglomerate stiffness**. Young's modulus of the macroscopic scaffold material, *E*_congl_, in GPa, computed for varying macroporosity, $\phi_{\text{macro}}^{\text{congl}}=\left\{0.3,0.4,0.5\right\}$, and **(A)** varying microporosity, $\phi_{\text{micro}}^{\text{polyHA}}=\left[0.2,0.6\right]$, **(B)** varying mesoporosity, $\phi_{\text{meso}}^{\text{gran}}=\left[0.1,0.3\right]$, **(C)** varying crack density, ϵ = [0, 25], and **(D)** varying bone tissue volume fraction, $f_{\text{bone}}^{\text{congl}}=\left[0,0.5\right]$.

Accordingly performed stiffness homogenization reveals that the Young's modulus of the scaffold material decreases non-linearly with increasing microporosity, whereas this dependence is the more pronounced the lower the macroporosity, see Figure 5A. Likewise, increasing the mesoporosity leads to a decreasing Young's modulus of the scaffold material, and the decrease is again more significant for lower macroporosities, see Figure 5B. Comparing Figures 5A,B suggests that the influence of the microporosity on the Young's modulus of the scaffold material is more significant than the influence of the mesoporosity—however, it should be noted that the technologically relevant range considered in these parameter studies is much larger for the microporosity than for the mesoporosity. An increase of the density of cracks obviously leads to a decreasing stiffness, see Figure 5C. Particularly for low crack densities an increase of the crack density parameter leads to a very steep stiffness loss. For higher crack density parameters, the additional stiffness loss due to further crack densification appears to be far less substantial. Notably, in the parameter study presented in Figure 5C, crack density parameters up to ϵ = 100 were considered. However, due to the minor dependence of the scaffold material stiffness on ϵ at high crack densities, only the range ϵ = {0, 25} is shown in Figure 5C. Finally, Figure 5D shows the dependence of *E*_congl_ on the bone tissue volume fraction. Obviously, bone ingrowth leads to a stiffness increase of the scaffold material. Depending on the initial value of the macroporosity, the stiffness increase stops once all of the macropore space is filled with bone tissue. Thus, while the stiffness for $\phi_{\text{macro}}^{\text{congl}}=0.5$ is quasi-zero and thus much lower than for $\phi_{\text{macro}}^{\text{congl}}=0.3$, the opposite is true once all of the pore space is filled with bone tissue.

Importantly, all dependencies discussed so far are significantly non-linear, and also highly interrelated; e.g., the influence of an increasing crack density differs, in quantitative terms, between an uncracked and a strongly cracked material, and so forth. This implies that for being able to adequately estimate the stiffness of a hierarchical material such as the one studied in this paper, using simplistic empirical relations is certainly not expedient, and may lead to serious misestimations.

### 3.2. Comments on experimental validation

The micromechanical representations depicted in Figure 1, and the corresponding homogenization steps, have undergone extensive experimental validation, in the context of various material systems. The self-consistent scheme with needle-shaped solid phases oriented in all space directions and spherical pores, see Section 2.2, has been experimentally validated for various porous hydroxapatite biomaterial systems (Peelen et al., 1978; Akao et al., 1981; de With et al., 1981; Shareef et al., 1993; Arita et al., 1995; Martin and Brown, 1995; Liu, 1998; Charrière et al., 2001; Fritsch et al., 2009); and it has been also corroborated for a wide range of other porous polycrystalline systems, such as gypsum (Sanahuja et al., 2010), or a variety of piezoelectric ceramics, alumina-, circonia-, and silicon-based materials (Fritsch et al., 2013).

The relevance of the Mori-Tanaka estimate for the mesoporous, cracked matrix-inclusion material system (of Section 2.3) can be readily seen from comparison of corresponding results to those of the Finite Element (FE) simulations of Dejaco et al. (2012): μCT scans of the investigated granule reveal a microporosity of $\phi_{\text{micro}}^{\text{polyHA}}=0.445$, a mesoporosity of $\phi_{\text{meso}}^{\text{gran}}=0.189$, a crack number of *N*≈3.80 × 10^−6^ μm^−3^, and an average crack radius of *r*_cr_ ≈ 260 μm; the latter two yielding a crack density parameter of ϵ_avg_ = 66.85. Inserting this compositional data into the stiffness estimate of Equation (3) yields a homogenized shear modulus of $\mu_{\text{gran}}^{\text{hom}}=78.50$ MPa. It needs to be compared to the shear modulus corresponding to the FE-modeled splitting test of Dejaco et al. (2012), which can be retrieved by the analytical formula for a sphere loaded at its poles (Lurje, 1963). The corresponding shear modulus amounts to $\mu_{\text{gran}}^{\text{FE}}=80.63$ MPa. The good agreement between $\mu_{\text{gran}}^{\text{hom}}$ and $\mu_{\text{gran}}^{\text{FE}}$ impressively underlines the reliability of the homogenization scheme defined by Equation (3).

The micromechanics of granular assemblies as seen in the top image of Figure 1 are standardly treated by the self-consistent scheme, as has been validated for material systems such as shale (Ortega et al., 2007). This renders the choice of a self-consistent assembly of pores and coated spheres as described in Sections 2.4 and 2.5 as very natural. Of course, an additional direct mechanical testing of bone-scaffold compounds is of general interest—however, given various technological and ethical challenges, this is clearly beyond the scope of this manuscript. Our philosophy is rather to collect and integrate, by use of latest engineering science developments, the large existing data base in biomaterial science and beyond, in order to bring forth novel design solutions for tissue engineering.

### 3.3. Toward mathematical modeling-based biomaterial design

**Figure 6:**
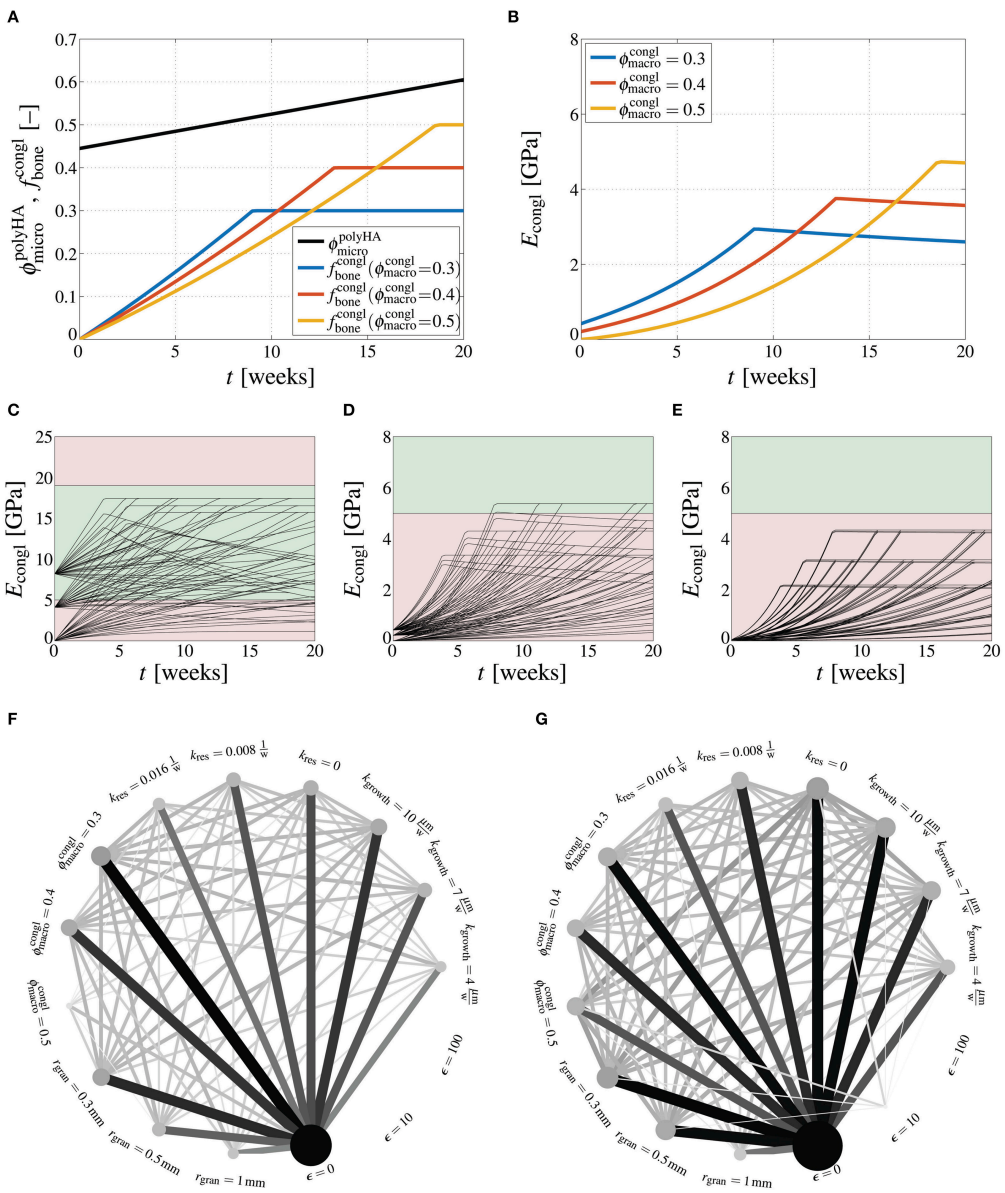
**How design parameter combinations affect bone regeneration**. Evaluation of bone regeneration kinetics laws, Equations (59) and (60), for *r*_gran_ = 500 μm, ϵ = 10, *k*_growth_ = 7 μm/week, and *k*_res_ = 0.008 week^−1^, in terms of microporosity and bone tissues volume fraction evolutions **(A)**, and in terms of the respective Young's modulus **(B)**; model-predicted development of the Young's modulus of the macroscopic scaffold material, *E*_congl_, in GPa, during bone regeneration, for varying crack density parameter ϵ = {0, 10, 100}, granule radius *r*_gran_ = {300 μm, 500 μm, 1000 μm}, macroporosity $\phi_{\text{macro}}^{\text{congl}}=\left\{0.3,0.4,0.5\right\}$, resorption rate $k_{\text{res}}=\left\{0,0.008{\text{week}}^{-1},0.016{\text{week}}^{-1}\right\}$, and bone ingrowth rate *k*_growth_ = {4 μm/week, 7 μm/week, 10 μm/week}, depicted for **(C)** ϵ = 0, **(D)** ϵ = 10, and **(E)** ϵ = 100, with the green area ranging from 5 to 19 GPa indicating the targeted stiffness range; graphical representation of successful parameter combinations at **(F)** *t* = 5 weeks, and **(G)** *t* = 20 weeks, with circle sizes being proportional to the success of specific parameter combinations and line thicknesses being proportional to the success of specific parameter combinations.

Finally, we present an outlook on how the presented homogenization scheme could be utilized in biomaterial design. In particular, the important question of how the stiffness of the scaffold material, once implanted, evolves during bone regeneration is addressed—notably, bone regeneration involves growth of new bone tissue, with rate *k*_growth_, and resorption of hydroxyapatite crystals, with rate *k*_res_, see Section 2.6. First, the development of the material's macroscopic Young's modulus is studied when considering a specific set of parameters, namely, *r*_gran_ = 500 μm, ϵ = 10, *k*_growth_ = 7 μm/week, and *k*_res_ = 0.008week^−1^. Corresponding evaluation of the bone regeneration kinetics laws considered in this work, see Equations (59) and (60), shows that the development of the bone tissue volume fraction depends on the macroporosity, while the resorption-related increase of the microporosity depends solely on the resorption rate, see Figure 6A. The Young's modulus increases due to the addition of new bone tissue in the macroscopic pore space up to the point where all pore space is occupied by bone tissue, after which the Young's modulus slightly decreases due to still progressing resorption of hydroxyapatite, see Figure 6B. Thus, although at *t* = 0 the macroscopic stiffness increases with decreasing macroporosity, the eventual stiffness after filling up the macroscopic pore space by new bone tissue is actually proportional to the macroporosity.

Extending the elucidation of how the macroscopic stiffness is influenced by the various design parameters of the scaffold material leads actually to a multi-factorial task. Here, we consider the following parameter variations: ϵ = {0, 10, 100}, *r*_gran_ = {300 μm, 500 μm, 1000 μm}, $\phi_{\text{macro}}^{\text{congl}}=\left\{0.3,0.4,0.5\right\}$, $k_{\text{res}}=\left\{0,0.008{\text{week}}^{-1},0.016{\text{week}}^{-1}\right\}$, *k*_growth_ = {4 μm/week, 7 μm/week, 10 μm/week}. Then, the arising 243 parameter combinations yield as many corresponding developments of the Young's modulus *E*_congl_ over time, see Figures 6C–E. Across the human mandible, for which the here studied scaffold material has been developed, a significant variation of the apparent density of the bone organ, and consequently of the stiffness has been observed (Kingsmill and Boyde, 1998; Swasty et al., 2009; Daegling et al., 2011). E.g., in (Daegling et al., 2011), the Young's modulus of mandibular bone is revealed to vary between 5 and 19 GPa—this range of targeted stiffness is indicated in Figures 6C, D by green color, whereas too low or two high stiffness are indicated by red color. It is instructive to evaluate which specific parameter values and which specific parameter combinations lead to the required scaffold stiffness within a reasonable duration. Figures 6F,G show, on the one hand, how often (out of the respectively possible 81 parameter combinations) a specific parameter value has led to a stiffness within the targeted range—the larger (and the darker) the circles related to a specific parameter value, the more often this parameter has led to an appropriate Young's modulus. On the other hand, the lines between two specific parameters indicate how often a specific parameter combination was successful—the thicker (and the darker) the lines, the more often this parameter combination has led to an appropriate Young's modulus. It is striking that after 5 weeks of bone regeneration, the crack density is the by far dominating parameter, i.e., only uncracked materials allow to reach the targeted stiffness, while all other parameters appear to be of minor importance, see Figure 6F. After 20 weeks, however, a low crack density still plays an important role, but also a low granule radius (*r*_gran_ = 300 μm), a low resorption rate (*k*_res_ = 0), and a high bone growth rate (*k*_growth_ = 10 μm/week) turn out as essential for the stiffness development of the scaffold material. In terms of parameter combinations, particularly the combination of ϵ = 0 and *k*_res_ = 0 turns out as crucial factor for the stiffness development.

**Figure 7:**
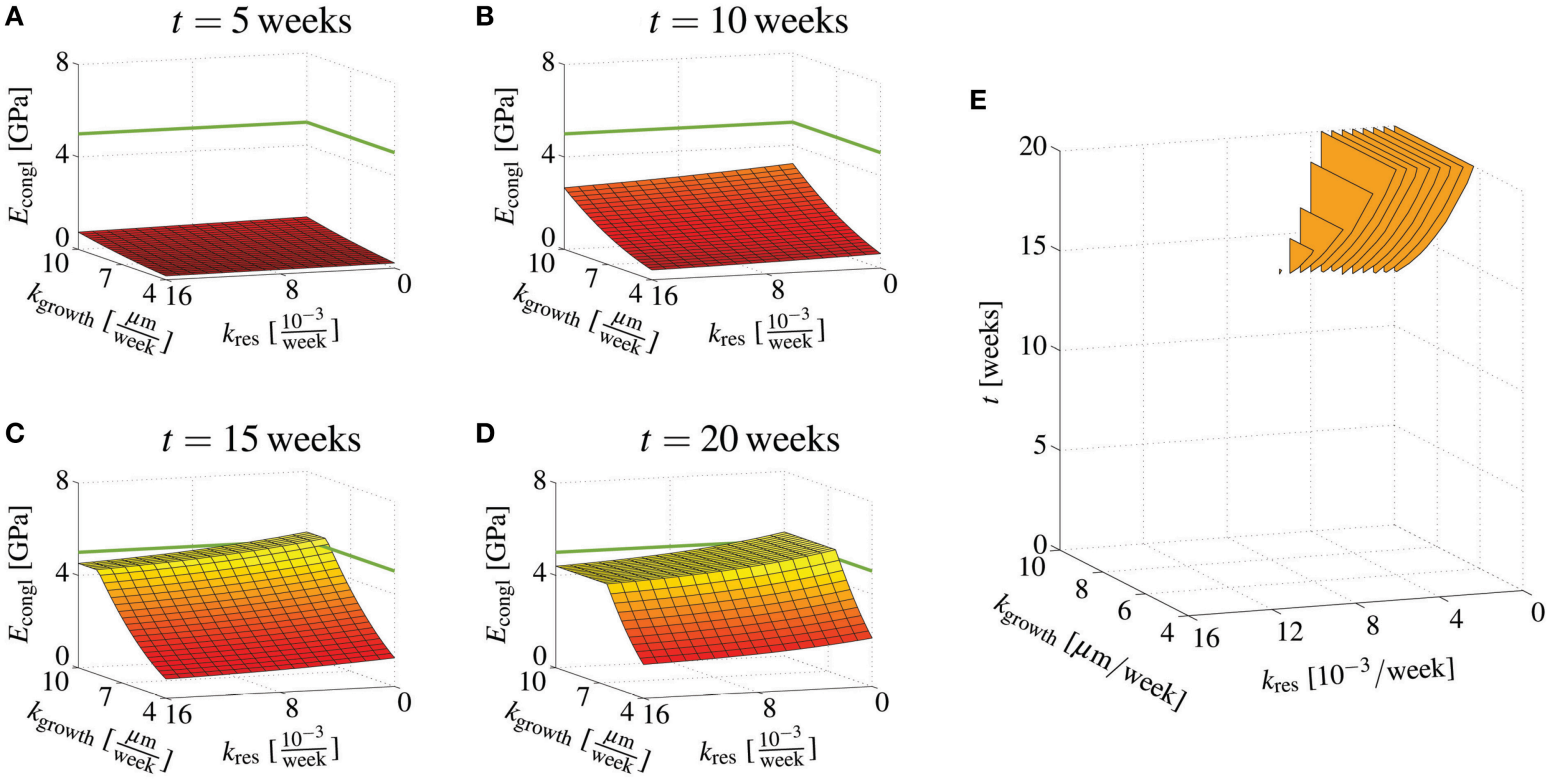
**Effects of bone regeneration kinetics parameters on the overall conglomerate stiffness at distinct time points**. Young's modulus of the macroscopic scaffold material for ϵ = 10, $\phi_{\text{macro}}^{\text{congl}}=0.4$, and *r*_gran_ = 500 μm, as well as $k_{\text{res}}=\left[0,0.016{\text{week}}^{-1}\right]$ and *k*_growth_ = [4 μm/week, 10 μm/week], after **(A)** 5 days, **(B)** 10 days, **(C)** 15 days, and **(D)** 20 days; **(E)** illustration when certain combinations of *k*_res_ and *k*_form_ yield 5GPa ≤ *E*_congl_ ≤ 19GPa.

Considering that the scaffold resorption rate and the bone ingrowth rate are kind of tunable properties, e.g., by slightly changing the chemical composition of the hydroxyapatite needles, or by adding bone morphogenetic proteins, it is interesting to study the stiffness development for continuous variations of these two quantities. For this purpose, a scaffold material is studied with ϵ = 10, $\phi_{\text{macro}}^{\text{congl}}=0.4$ and *r*_gran_ = 500 μm, whereas *k*_res_ and *k*_growth_ are varied in the ranges of $k_{\text{res}}=\left[0,0.016{\text{week}}^{-1}\right]$ and *k*_growth_ = [4 μm/week, 10 μm/week]. Then, our model suggests that after 5 weeks none of the considered combinations of *k*_res_ and *k*_growth_ leads to *E*_congl_ ≥ 5 GPa, that after 10 weeks, a combination of low resorption rate and high ingrowth rate leads to sufficient stiffness, and that after 20 weeks virtually all bone growth rates (within the considered range) lead to sufficient stiffness as long as the bone resorption rate remains at a low level, see Figures 7A–D. Figure 7E shows at which time instants *E*_congl_ ≥ 5 GPa is reached for specific combination of *k*_res_ and *k*_growth_. Similar computations can be performed for other combinations of ϵ, *r*_gran_, and $\phi_{\text{macro}}^{\text{congl}}$, in order to work out the appropriate parameter space.

Actually utilizing the stiffness prediction tool proposed in this paper for scaffold optimization, requires however more detailed knowledge on the bone ingrowth and scaffold resorption kinetics. In the here presented studies, linear kinetics laws were assumed. In the physiological environment of the implanted scaffold material, long-term occurrence of such linear behavior is however unlikely, for several reasons: Bone ingrowth is a process which typically occurs in degressive fashion, see e.g., (Hing et al., 2004; Cancedda et al., 2007); on the one hand because of purely geometric reasons, namely due to decreasing area on which new bone tissue can be laid down, and on the other hand because of the decreasing availability (with decreasing porosity) of the biological factors necessary for initiating bone apposition. Also, specifically designing a scaffold, for a particular patient, requires information on the location of insertion, e.g., density distributions gained from CT imaging may be sufficient to estimate the site-specific stiffness distribution (Hellmich et al., 2008). Considering these additional influences, namely both the biological environment and the mechanical environment which are to be expected *in vivo*, is however beyond the scope of this paper.

## 4. Conclusions

In this paper, we have presented a new mathematical model for determining the stiffness of a hydroxyapatite-based granular biomaterial, through upscaling of compositional and morphological information known on three distinct observation scales. The model predictions are, from a qualitative point of view, plausible. The distinctively non-linear dependencies of the derived elastic constants on underlying nano-, micro-, and macro-porosities, as well as on the extent of cracks emerging on the micro-scale underline the necessity of using sophisticated mathematical models when reliable stiffness estimates are required.

The proposed model provides substantiated outlooks as to how specific design parameters influence the macroscopic stiffness of the scaffold-bone compound. In order to reach a physiologically relevant Young's modulus of mandibular bone, typically ranging from 5 to 19 GPa (Daegling et al., 2011), granules with the lowest possible crack density need to be produced, especially at early stages of bone regeneration. Later, other design parameters, such as a low granule radius or a high bone formation rate become relevant as well. In such a way, as demonstrated in Section 3.3, optimal design parameters or even combinations of design parameters can be worked out, paving the way to mathematical modeling-driven optimization of the scaffold-bone compound's performance.

Furthermore, we plan to couple the here presented mechanical model with computed tomography (CT) image-to-mechanical properties conversion techniques, as demonstrated for different materials in (Scheiner et al., 2009; Dejaco et al., 2012). Such conversion techniques allow for direct interpretation of the gray value distributions that constitute CT images in terms of the corresponding distributions of mechanical properties. Finally, once such three-dimensional distributions of the scaffold stiffness and the surrounding bone matrix are computed, the model could be further coupled to recently developed mechanobiology models (Scheiner et al., 2013), eventually allowing to assess the bone regeneration progress for prescribed mechanical loading regimes. These model upgrades will allow to design patient-specific scaffold materials and structures, which is believed to entail a significant increase of the efficiency of such materials.

## Author contributions

SS: Conceptual model design, model implementation, performance of all numerical studies, interpretation of the results, as well as drafting and all tasks related to finalizing the manuscript. CH: Conceptual model design, interpretation of the results, and all tasks related to finalizing the manuscript. VK: Processing of the studied material, contributions to interpretation of the experimental results, final proofreading of the manuscript. AG: Implementation of histological and dissolution studies, final proofreading of the manuscript.

### Conflict of interest statement

The authors declare that the research was conducted in the absence of any commercial or financial relationships that could be construed as a potential conflict of interest.

## References

[B1] Akao M. Aoki H. Kato K. (1981). Mechanical properties of sintered hydroxyapatite for prosthetic applications. J. Mater. Sci. 16, 809–812. 10.1007/BF02402799

[B2] Arita I. Wilkinson D. Mondragón M. Castaño V. (1995). Chemistry and sintering behaviour of thin hydroxyapatite ceramics with controlled porosity. Biomaterials 16, 403–408. 10.1016/0142-9612(95)98858-B 7662826

[B3] Ashman R. van Buskirk W. (1987). The elastic properties of a human mandible. Adv. Dent. Res. 1, 64–67. 3326617 10.1177/08959374870010011401

[B4] Benveniste Y. (1987). A new approach to the application of Mori-Tanaka's theory in composite materials. Mech. Mater. 6, 147–157. 10.1016/0167-6636(87)90005-6

[B5] Bertrand E. Hellmich C. (2009). Multiscale elasticity of tissue engineering scaffolds with tissue-engineered bone: a continuum micromechanics approach. J. Eng. Mech. 135, 395–412. 10.1061/(ASCE)0733-9399(2009)135:5(395)

[B6] Budianksy B. O'Connell R. (1976). Elastic moduli of a cracked solid. Int. J. Solids Struc. 12, 81–97.

[B7] Cancedda R. Cedola A. Giuliani A. Komlev V. Lagomarsino S. Mastrogiacomo M.. (2007). Bulk and interface investigations of scaffolds and tissue-engineered bones by X-ray microtomography and X-ray microdiffraction. Biomaterials 28, 2505–2524. 10.1016/j.biomaterials.2007.01.022 17292959

[B8] Charrière E. Terrazzoni S. Pittet C. Mordasini P. Dutoit M. Lemaître J.. (2001). Mechanical characterization of brushite and hydroxyapatite cements. Biomaterials 22, 2937–2945. 10.1016/S0142-9612(01)00041-2 11561900

[B9] Czenek A. Blanchard R. Dejaco A. Sigurjónsson O. Örlygsson G. Gargiulo P. (2014). Quantitative intravoxel analysis of microct-scanned resorbing ceramic biomaterials – perspectives for computer-aided biomaterial design. J. Mater. Res. 29, 2757–2772. 10.1557/jmr.2014.326

[B10] Daegling D. Granatosky M. McGraw W. Rapoff A. (2011). Spatial patterning of bone stiffness variation in the colobine alveolar process. Arch. Oral Biol. 56, 220–230. 10.1016/j.archoralbio.2010.10.008 21055726

[B11] de With G. van Dijk H. Hattu N. Prijs K. (1981). Preparation, microstructure and mechanical properties of dense polycrystalline hydroxy apatite. J. Mater. Sci. 16, 1592–1598. 10.1007/BF02396876

[B12] Dejaco A. Komlev V. Jaroszewicz J. Swieszkowski W. Hellmich C. (2012). Micro CT-based multiscale elasticity of double-porous (pre-cracked) hydroxyapatite granules for regenerative medicine. J. Biomech. 45, 1068–1075. 10.1016/j.jbiomech.2011.12.026 22296936

[B13] Deudé V. Dormieux L. Kondo D. Maghous S. (2002). Micromechanical approach to nonlinear poroelasticity: application to cracked rocks. J. Eng. Mech. 128, 848–855. 10.1061/(ASCE)0733-9399(2002)128:8(848)

[B14] Dormieux L. Lemarchand E. Kondo D. Fairbairn E. (2004). Elements of poro-micromechanics applied to concrete. Mater. Struct. Concrete Sci. Eng. 37, 31–42. 10.1617/14099

[B15] Drugan W. Willis J. (1996). A micromechanics-based nonlocal constitutive equation and estimates of representative volume element size for elastic composites. J. Mech. Phys. Solids 44, 497–524. 10.1016/0022-5096(96)00007-5

[B16] Eshelby J. (1957). The determination of the elastic field of an ellipsoidal inclusion, and related problems. Proc. R. Soc. Lon. Ser. A 241, 376–396.

[B17] Fritsch A. Dormieux L. Hellmich C. (2006). Porous polycrystals built up by uniformly and axisymmetrically oriented needles: homogenization of elastic properties. C R Mécanique 334, 151–157. 10.1016/j.crme.2006.01.008

[B18] Fritsch A. Dormieux L. Hellmich C. Sanahuja J. (2009). Mechanical behavior of hydroxyapatite biomaterials: an experimentally validated micromechanical model for elasticity and strength. J. Biomed. Mater. Res. A 88A, 149–161. 10.1002/jbm.a.31727 18286602

[B19] Fritsch A. Hellmich C. (2007). “Universal” microstructural patterns in cortical and trabecular, extracellular and extravascular bone materials: micromechanics-based prediction of anisotropic elasticity. J. Theor. Biol. 244, 597–620. 10.1016/j.jtbi.2006.09.013 17074362

[B20] Fritsch A. Hellmich C. Young P. (2013). Micromechanics-derived scaling relations for poroelasticity and strength of brittle porous polycrystals. J. Appl. Mech. 80:020905 10.1115/1.4007922

[B21] Gilmore R. Katz J. (1982). Elastic properties of apatites. J. Mater. Sci. 17, 1131–1141. 10.1007/BF00543533

[B22] Hamed E. Lee Y. Jasiuk I. (2010). Multiscale modeling of elastic properties of cortical bone. Acta Mech. 213, 131–154. 10.1007/s00707-010-0326-5

[B23] Hart R. Hennebel V. Thongpreda N. Van Buskirk W. Anderson R. (1992). Modeling the biomechanics of the mandible: a three-dimensional finite element study. J. Biomech. 25, 261–286. 10.1016/0021-9290(92)90025-V 1564061

[B24] Hellmich C. Barthélémy J.-F. Dormieux L. (2004a). Mineral-collagen interactions in elasticity of bone ultrastructure - a continuum micromechanics approach. Eur. J. Mech. A/Solids 23, 783–810. 10.1016/j.euromechsol.2004.05.004

[B25] Hellmich C. Kober C. Erdmann B. (2008). Micromechanics-based conversion of CT data into anisotropic elasticity tensors, applied to FE simulations of a mandible. Ann. Biomed. Eng. 36, 108–122. 10.1007/s10439-007-9393-8 17952601

[B26] Hellmich C. Ulm F.-J. (2002). Micromechanical model for ultra-structural stiffness of mineralized tissues. J. Eng. Mech. 128, 898–908. 10.1061/(ASCE)0733-9399(2002)128:8(898)

[B27] Hellmich C. Ulm F.-J. Dormieux L. (2004b). Can the diverse elastic properties of trabecular and cortical bone be attributed to only a few tissue-independent phase properties and their interactions? Arguments from a multiscale approach. Biomech. Model. Mechanobiol. 2, 219–238. 10.1007/s10237-004-0040-0 15054639

[B28] Hervé E. Zaoui A. (1993). n-Layered inclusion-based micromechanical modelling. Int. J. Eng. Sci. 31, 1–10. 10.1016/0020-7225(93)90059-4

[B29] Hill R. (1963). Elastic properties of reinforced solids: some theoretical principles. J. Mech. Phys. Solids 11, 357–372. 10.1016/0022-5096(63)90036-X

[B30] Hing K. Best S. Tanner K. Bonfield W. Revell P. (2004). Mediation of bone ingrowth in porous hydroxyapatite bone graft substitutes. J. Biomed. Mater. Res. A 68, 187–200. 10.1002/jbm.a.10050 14661264

[B31] Katz J. Ukraincik K. (1971). On the anisotropic elastic properties of bone. Calcif. Tissue Int. 4, 221–227. 10.1016/0021-9290(71)90007-8 5119417

[B32] Kingsmill V. Boyde A. (1998). Variation in the apparent density of human mandibular bone with age and dental status. J. Anat. 192, 233–244. 10.1046/j.1469-7580.1998.19220233.x 9643424 PMC1467757

[B33] Kohlhauser C. Hellmich C. (2013). Ultrasonic contact pulse transmission for elastic wave velocity and stiffness determination: influence of specimen geometry and porosity. Eng. Struct. 47, 115–133. 10.1016/j.engstruct.2012.10.027

[B34] Komlev V. Barinov S. Girardin E. Oscarsson S. Rosengren A. Rustichelli F. (2003). Porous spherical hydroxyapatite and fluorhydroxyapatite granules: processing and characterization. Sci. Technol. Adv. Mater. 4, 503–508. 10.1016/j.stam.2003.11.007

[B35] Komlev V. Barinov S. Koplik E. (2002). A method to fabricate porous spherical hydroxyapatite granules intended for time-controlled drug release. Biomaterials 23, 3449–3454. 10.1016/S0142-9612(02)00049-2 12099288

[B36] Koritoth T. Versluis A. (1997). Modeling the mechanical behavior of the jaws and their related structures by Finite Element (FE) analysis. Crit. Rev. Oral Biol. Med. 8, 90–104. 10.1177/10454411970080010501 9063627

[B37] Liu D.-M. (1998). Preparation and characterisation of porous hydroxyapatite bioceramic via slip-casting route. Ceram. Int. 24, 441–446. 10.1016/S0272-8842(97)00033-3

[B38] Lurje A. (1963). Räumliche Probleme der Elastizitätstheorie [Spatial Problems of Elasticity Theory]. Berlin: Akademie Verlag.

[B39] Martin R. Brown P. (1995). Mechanical properties of hydroxyapatite formed at physiological temperatures. J. Mater. Sci. Mater. Med. 6, 138–143. 10.1007/BF00120289

[B40] Mori T. Tanaka K. (1973). Average stress in matrix and average elastic energy of materials with misfitting inclusions. Acta Metallurg. 21, 571–574. 10.1016/0001-6160(73)90064-3

[B41] Ortega J. Ulm F.-J. Abousleiman Y. (2007). The effect of the nanogranular nature of shale on their poroelastic behavior. Acta Geotech. 2, 155–182. 10.1007/s11440-007-0038-8

[B42] Peelen J. Rejda B. de Groot K. (1978). Preparation and properties of sintered hydroxylapatite. Ceramurgia Int. 4, 71–74. 10.1016/0390-5519(78)90122-9

[B43] Pichler B. Hellmich C. Eberhardsteiner J. (2009). Spherical and acicular representation of hydrates in a micromechanical model for cement paste: prediction of early-age elasticity and strength. Acta Mech. 203, 137–162. 10.1007/s00707-008-0007-9

[B44] Salençon J. (2001). Handbook of Continuum Mechanics. Berlin; Heidelberg: Springer-Verlag.

[B45] Sanahuja J. Dormieux L. Meille S. Hellmich C. Fritsch A. (2010). Micromechanical explanation of elasticity and strength of gypsum: from elongated anisotropic crystals to isotropic porous polycrystals. J. Eng. Mech. 136, 239–253. 10.1061/(ASCE)EM.1943-7889.0000072

[B46] Scheiner S. Pivonka P. Hellmich C. (2013). Coupling systems biology with multiscale mechanics, for computer simulations of bone remodeling. Comput. Methods Appl. Mech. Eng. 254, 181–196. 10.1016/j.cma.2012.10.015

[B47] Scheiner S. Pivonka P. Hellmich C. (2016). Poromicromechanics reveals that physiological bone strains induce osteocyte-stimulating lacunar pressure. Biomech. Model. Mechanobiol. 15, 9–28. 10.1007/s10237-015-0704-y 26220453 PMC4779462

[B48] Scheiner S. Sinibaldi R. Pichler B. Komlev V. Renghini C. Vitale-Brovarone C.. (2009). Micromechanics of bone tissue-engineering scaffolds, based on resolution error-cleared computer tomography. Biomaterials 30, 2411–2419. 10.1016/j.biomaterials.2008.12.048 19135717

[B49] Shareef M. Messer P. van Noort R. (1993). Fabrication, characterization and fracture study of a machinable hydroxyapatite ceramic. Biomaterials 14, 69–75. 10.1016/0142-9612(93)90078-G 8381034

[B50] Stroud A. (1971). Approximate Calculation of Multiple Integrals. Englewood Cliffs, NJ: Prentice-Hall.

[B51] Suquet P. (1997). Continuum Micromechanics, volume 377 of CISM Courses and Lectures. Wien; New York, NY: Springer Verlag.

[B52] Swasty D. Lee J. Huang J. Maki K. Gansky S. Hatcher S.. (2009). Anthropometric analysis of the human mandibular cortical bone as assessed by cone-beam computed tomography. J. Oral Maxillofac. Surg. 67, 491–500. 10.1016/j.joms.2008.06.089 19231771

[B53] Thompson M. Willis J. (1991). A reformation of the equations anisotropic elasticity. J. Appl. Mech. 58, 612–616. 10.1115/1.2897239

[B54] van Ruijven L. Mulder L. van Eijden T. (2007). Variations in mineralization affect the stress and strain distributions in cortical and trabecular bone. J. Biomech. 40, 1211–1218. 10.1016/j.jbiomech.2006.06.004 16934818

[B55] Zaoui A. (1997). Structural Morphology and Constitutive Behavior of Microheterogeneous Materials. Wien; New York, NY: Springer-Verlag.

[B56] Zaoui A. (2002). Continuum micromechanics: survey. J. Eng. Mech. 128, 808–816. 10.1061/(ASCE)0733-9399(2002)128:8(808)

