# Supplementary material for: Multiscale Mathematical Modeling in Dental Tissue Engineering: Toward Computer-Aided Design of a Regenerative System Based on Hydroxyapatite Granules, Focussing on Early and Mid-Term Stiffness Recovery
Source: Front Physiol. 2016 Sep 21;7:383. doi: 10.3389/fphys.2016.00383 (PMC5030309; doi:10.3389/fphys.2016.00383)
Supplement: Supplementary file 1 [file DataSheet1.PDF]

Supplementary Material: Multiscale mathematical modeling in dental tissue engineering: towards computer-aided design of a regenerative system based on hydroxyapatite granules, focusing on early and mid-term stiffness recovery

Stefan Scheiner [1,*], Vladimir S. Komlev [2,3], Alexey N. Gurin [4] and Christian Hellmich [1]

[1] *Institute for Mechanics of Materials and Structures, TU Wien – Vienna University of Technology, Vienna, Austria*
[2] *A.A. Baikov Institute of Metallurgy and Materials Science, Russian Academy of Sciences, Moscow, Russia*
[3] *Institute of Laser and Information Technologies, Russian Academy of Sciences, Moscow, Russia*
[4] *Central Scientific Research Institute of Dentistry and Maxillofacial Surgery, Moscow, Russia*

Correspondence*:
Stefan Scheiner
Institute for Mechanics of Materials and Structures, TU Wien – Vienna University of Technology, Karlsplatz 13/202, A-1040 Vienna, Austria,
stefan.scheiner@tuwien.ac.at

Dental bioengineering and mathematical modeling

OVERVIEW

1 Hill and Eshelby tensors . . . 2
1.1 General case . . . 2
1.2 Cylindrical inclusions in an isotropic matrix . . . 2
1.3 Spherical inclusions in an isotropic matrix . . . 3

2 Derivation of the macroscopic bulk modulus of the scaffold-bone conglomerate . . . 3
2.1 Material constants . . . 3
2.2 Coefficients for definition of the strain field for volumetric loading . . . 3
2.3 Volume averages of strain and stress fields in granule and bone phases for volumetric loading . . . 4

3 Derivation of the macroscopic shear modulus of the scaffold-bone conglomerate . . . 5
3.1 Derivation of governing differential equations . . . 5
3.2 Material constants . . . 5
3.3 Strain and stress fields for deviatoric loading, and the governing coefficients . . . 6
3.4 Volume averages of strain and stress fields in granule and bone phases for deviatoric loading . . . 8

1 HILL AND ESHELBY TENSORS

1.1 GENERAL CASE

Following the work of Laws (1977, 1985), the components of the fourth-order Hill tensor relating to an ellipsoidal inclusion in an anisotropic matrix read, in the notation Hellmich et al. (2004), as

$$P_{ijkl} = \frac{1}{16\pi\alpha^{1/2}} \int_{\Omega} \frac{1}{t^3} \left[\hat{g}_{il} w_j w_k + \hat{g}_{ik} w_j w_l + \hat{g}_{jl} w_i w_k + \hat{g}_{jk} w_i w_l\right] \, \mathrm{d}S(\mathrm{w})\,. \tag{1}$$

In Eq. (1), $\alpha = \det \alpha_{ij}$, relating to the equation of an ellipsoid, $\alpha_{ij} x_i x_j = 1$, considers the shape of the ellipsoidal inclusion; $\mathrm{d}S(\mathbf{w})$ is a surface element on the unit sphere, the total surface area being Ω; w_1, w_2, and w_3 are the components of the unit length vector $\mathbf{w}$ oriented from the origin of the unit sphere to the surface element $\mathrm{d}S(\mathbf{w})$; t is defined through $t = \sqrt{(w_i w_j)/\alpha_{ij}}$; and $\hat{g}_{ik}$ are the components of the inverse of the second-order tensor $(C^0_{ijkl} w_j w_l)$, with C^0_{ijkl} as the components of the stiffness tensor of the anisotropic matrix. Furthermore, the unit vector $\mathbf{w}$ can be expressed in spherical (Euler) coordinates $\vartheta \in [0,\, \pi]$ and $\varphi \in [0,\, 2\pi]$, namely $w_1 = \sin\vartheta \cos\varphi$, $w_2 = \sin\vartheta \sin\varphi$, $w_3 = \cos\vartheta$, implying $\mathrm{d}S(\mathbf{w}) = \sin\vartheta \, \mathrm{d}\varphi \, \mathrm{d}\vartheta$.

1.2 CYLINDRICAL INCLUSIONS IN AN ISOTROPIC MATRIX

The non-zero components of the fourth-order tensor $\mathbb{S}^{\mathrm{iso}}_{\mathrm{cyl}}$ relating to a cylindrical inclusion in an isotropic matrix read as Eshelby (1957)

$$S^{\mathrm{iso}}_{\mathrm{cyl},1111} = S^{\mathrm{iso}}_{\mathrm{cyl},2222} = \frac{9}{4} \frac{k_{\mathrm{iso}} + \mu_{\mathrm{iso}}}{3k_{\mathrm{iso}} + 4\mu_{\mathrm{iso}}} \tag{2}$$

$$S^{\mathrm{iso}}_{\mathrm{cyl},1122} = S^{\mathrm{iso}}_{\mathrm{cyl},2211} = \frac{1}{4} \frac{3k_{\mathrm{iso}} - 5\mu_{\mathrm{iso}}}{3k_{\mathrm{iso}} + 4\mu_{\mathrm{iso}}} \tag{3}$$

$$S^{\mathrm{iso}}_{\mathrm{cyl},1133} = S^{\mathrm{iso}}_{\mathrm{cyl},3311} = \frac{1}{2} \frac{3k_{\mathrm{iso}} - 2\mu_{\mathrm{iso}}}{3k_{\mathrm{iso}} + 4\mu_{\mathrm{iso}}} \tag{4}$$

$$S^{\mathrm{iso}}_{\mathrm{cyl},1212} = S^{\mathrm{iso}}_{\mathrm{cyl},2121} = \frac{1}{4} \frac{3k_{\mathrm{iso}} + 7\mu_{\mathrm{iso}}}{3k_{\mathrm{iso}} + 4\mu_{\mathrm{iso}}} \tag{5}$$

$$S^{\mathrm{iso}}_{\mathrm{cyl},1313} = S^{\mathrm{iso}}_{\mathrm{cyl},3131} = S^{\mathrm{iso}}_{\mathrm{cyl},2323} = S^{\mathrm{iso}}_{\mathrm{cyl},3232} = \frac{1}{4}\,, \tag{6}$$

with k_{iso} and μ_{iso} as bulk and shear modulus of the isotropic matrix. The corresponding Hill tensor, $\mathbb{P}^{\mathrm{iso}}_{\mathrm{cyl}}$, follows from

$$\mathbb{P}^{\mathrm{iso}}_{\mathrm{cyl}} = \mathbb{S}^{\mathrm{iso}}_{\mathrm{cyl}} : (\mathbb{C}_{\mathrm{iso}})^{-1}\,, \tag{7}$$

where $\mathbb{C}_{\mathrm{iso}}$ is the isotropic stiffness tensor of the matrix. Note that $\mathbb{P}^{\mathrm{iso}}_{\mathrm{cyl}}$ can be derived through evaluation of Eq. (1). Eqs. (2) – (7) are employed for evaluating the Hill tensor for cylindrical inclusions in a microporous hydroxyapatite polycrystal, $\mathbb{P}^{\mathrm{polyHA}}_{\mathrm{cyl}}(\vartheta, \varphi)$, occurring in Eq. (1) of the main paper.

1.3 SPHERICAL INCLUSIONS IN AN ISOTROPIC MATRIX

The Eshelby tensor relating to spherical inclusions in an isotropic matrix, $\mathbb{S}^{\text{iso}}_{\text{sph}}$, is defined through (Eshelby, 1957; Zaoui, 2002)

$$\mathbb{S}^{\text{iso}}_{\text{sph}} = \alpha^{\text{iso}}\mathbb{K} + \beta^{\text{iso}}\mathbb{J}\,, \tag{8}$$

with

$$\alpha^{\text{iso}} = \frac{3k_{\text{iso}}}{3k_{\text{iso}} + 4\mu_{\text{iso}}} \qquad \text{and} \qquad \beta^{\text{iso}} = \frac{6(k_{\text{iso}} + 2\mu_{\text{iso}})}{5(3k_{\text{iso}} + 4\mu_{\text{iso}})}\,. \tag{9}$$

The corresponding Hill tensor, $\mathbb{P}^{\text{iso}}_{\text{sph}}$, follows, analogously to Eq. (7), from

$$\mathbb{P}^{\text{iso}}_{\text{sph}} = \mathbb{S}^{\text{iso}}_{\text{sph}} : (\mathbb{C}_{\text{iso}})^{-1}\,. \tag{10}$$

Evaluation of Eq. (1), specialized for the present inclusion geometry and matrix stiffness, allows to derive the same result. Here, Eqs. (8) – (10) serve for evaluating the Hill tensor for spherical inclusions in the microporous hydroxyapatite polycrystal, $\mathbb{P}^{\text{polyHA}}_{\text{sph}}$, see Eqs. (1) and (3) of the main paper, and the Eshelby tensor of spherical inclusions within the scaffold-bone conglomerate, $\mathbb{S}^{\text{congl}}_{\text{sph}}$, see Eqs. (40) and (56) of the main paper.

2 DERIVATION OF THE MACROSCOPIC BULK MODULUS OF THE SCAFFOLD-BONE CONGLOMERATE

2.1 MATERIAL CONSTANTS

Material constants $\mathcal{M}_i$, $\mathcal{N}_i$, $\mathcal{O}_i$, $\mathcal{P}_i$, introduced in Eqs. (27) – (29) of the main paper, are defined as

$$\mathcal{M}_i = C_{i,rrrr}\left(-\frac{1}{2} + n_i\right) + 2C_{i,rr\vartheta\vartheta}\,, \tag{11}$$

$$\mathcal{N}_i = C_{i,rrrr}\left(-\frac{1}{2} - n_i\right) + 2C_{i,rr\vartheta\vartheta}\,, \tag{12}$$

$$\mathcal{O}_i = C_{i,rr\vartheta\vartheta}\left(-\frac{1}{2} + n_i\right) + C_{i,\vartheta\vartheta\vartheta\vartheta} + C_{i,\vartheta\vartheta\varphi\varphi}\,, \tag{13}$$

$$\mathcal{P}_i = C_{i,rr\vartheta\vartheta}\left(-\frac{1}{2} - n_i\right) + C_{i,\vartheta\vartheta\vartheta\vartheta} + C_{i,\vartheta\vartheta\varphi\varphi}\,, \tag{14}$$

with n_i according to Eq. (22) of the main paper.

2.2 COEFFICIENTS FOR DEFINITION OF THE STRAIN FIELD FOR VOLUMETRIC LOADING

Displacement, strain, and stress fields found in Eqs. (24) – (29) of the main paper require determination of the six parameters $\Gamma^k_{i,j}$, ($i =$ gran, bone, congl; $j = 1, 2$). $\Gamma^k_{\text{gran},2}$ follows from evaluation of Eq. (23) of the main paper at $r = 0$, while considering the isotropy of the granule material (thus $n_{\text{gran}} = 3/2$), $\Gamma^k_{\text{gran},2} = 0$. $\Gamma^k_{\text{congl},1}$, on the other hand, follows from evaluation of Eq. (23) of the main paper at $r = \infty$, while considering the isotropy of the scaffold-bone conglomerate (thus $n_{\text{congl}} = 3/2$), as well as Eqs. (18) and (19) of the main paper, $\Gamma^k_{\text{congl},1} = E_{\text{vol},0}/3$. In order to determine the four remaining parameters,

continuity conditions at the interfaces between the granule and the bone domain, as well as between the bone-domain and the surrounding matrix are employed,

$$\xi_{\text{gran},r}(r_1) = \xi_{\text{bone},r}(r_1)\,, \tag{15}$$
$$\xi_{\text{bone},r}(r_2) = \xi_{\text{congl},r}(r_2)\,, \tag{16}$$
$$\sigma_{\text{gran},rr}(r_1) = \sigma_{\text{bone},rr}(r_1)\,, \tag{17}$$
$$\sigma_{\text{bone},rr}(r_2) = \sigma_{\text{congl},rr}(r_2)\,. \tag{18}$$

Substituting the displacement and stress fields as defined through Eqs. (24) – (29) of the main paper into Eqs. (15) – (18) yields a system of four linear equations, giving access to

$$\Gamma^k_{\text{gran},1} = E_{\text{vol},0}\overline{\Gamma^k_{\text{gran},1}}/\mathcal{D}_k\,, \tag{19}$$
$$\Gamma^k_{\text{bone},1} = E_{\text{vol},0}\overline{\Gamma^k_{\text{bone},1}}\,r_1^{3/2-n_{\text{bone}}}/\mathcal{D}_k\,, \tag{20}$$
$$\Gamma^k_{\text{bone},2} = E_{\text{vol},0}\overline{\Gamma^k_{\text{bone},2}}\,r_1^{3/2+n_{\text{bone}}}/\mathcal{D}_k\,, \tag{21}$$
$$\Gamma^k_{\text{congl},2} = E_{\text{vol},0}\overline{\Gamma^k_{\text{congl},2}}\,r_1^{3}/\mathcal{D}_k\,. \tag{22}$$

Eqs. (19) – (22) consider the following definitions:

$$\overline{\Gamma^k_{\text{gran},1}} = \mathcal{F}^{\frac{1}{2}+\frac{n_{\text{bone}}}{3}}\big(3k_{\text{congl}} - 4\mu_{\text{congl}}\big)\big(\mathcal{M}_{\text{bone}} - \mathcal{N}_{\text{bone}}\big)\,, \tag{23}$$
$$\overline{\Gamma^k_{\text{bone},1}} = \overline{\Gamma^k_{\text{gran},1}}\,\frac{3k_{\text{gran}} - \mathcal{N}_{\text{bone}}}{\mathcal{M}_{\text{bone}} - \mathcal{N}_{\text{bone}}}\,, \tag{24}$$
$$\overline{\Gamma^k_{\text{bone},2}} = \mathcal{F}^{\frac{1}{2}+\frac{n_{\text{bone}}}{3}}\big(\mathcal{M}_{\text{bone}} - 3k_{\text{gran}}\big)\big(3k_{\text{congl}} - 4\mu_{\text{congl}}\big)\,, \tag{25}$$
$$\overline{\Gamma^k_{\text{congl},2}} = \mathcal{F}\left[\mathcal{F}^{\frac{2n_{\text{bone}}}{3}}\big(3k_{\text{congl}} - \mathcal{M}_{\text{bone}}\big)\big(3k_{\text{gran}} - \mathcal{N}_{\text{bone}}\big) + \big(\mathcal{M}_{\text{bone}} - 3k_{\text{gran}}\big)\big(3k_{\text{congl}} - \mathcal{N}_{\text{bone}}\big)\right], \tag{26}$$

$$\mathcal{D}_k = 3\mathcal{F}^{\frac{2n_{\text{bone}}}{3}}\big(\mathcal{M}_{\text{bone}} + 4\mu_{\text{congl}}\big)\big(3k_{\text{gran}} - \mathcal{N}_{\text{bone}}\big) + 3\big(\mathcal{M}_{\text{bone}} - 3k_{\text{gran}}\big)\big(4\mu_{\text{congl}} + \mathcal{N}_{\text{bone}}\big)\,, \tag{27}$$

with parameter $\mathcal{F}$ based on the composition of the underlying RVE,

$$\mathcal{F} = 1 + \frac{f_{\text{bone}}}{f_{\text{gran}}}\,. \tag{28}$$

2.3 VOLUME AVERAGES OF STRAIN AND STRESS FIELDS IN GRANULE AND BONE PHASES FOR VOLUMETRIC LOADING

Application of Eq. (49) of the main paper for the granule phase and the bone phase, while considering the involved material constants as given by Eqs. (23) – (28), yields

$$\big\langle \varepsilon_{\text{vol}}(\mathbf{x})\big\rangle_{\text{gran}} = \frac{3\overline{\Gamma^k_{\text{gran},1}}}{\mathcal{D}_k}E_{\text{vol},0}\,, \tag{29}$$
$$\big\langle \varepsilon_{\text{vol}}(\mathbf{x})\big\rangle_{\text{bone}} = \frac{3E_{\text{vol},0}}{\mathcal{D}_k(1-\mathcal{F})}\left[\overline{\Gamma^k_{\text{bone},1}}\left(1 - \mathcal{F}^{\frac{1}{2}+\frac{n_{\text{bone}}}{3}}\right) + \overline{\Gamma^k_{\text{bone},2}}\left(1 - \mathcal{F}^{\frac{1}{2}-\frac{n_{\text{bone}}}{3}}\right)\right]. \tag{30}$$

Analogously, application of Eq. (50) of the main paper for the granule phase and the bone phase yields

$$\langle\sigma_{\mathrm{m}}(\mathbf{x})\rangle_{\mathrm{gran}} = \frac{3k_{\mathrm{gran}}\overline{\Gamma^k_{\mathrm{gran},1}}}{\mathcal{D}_k}E_{\mathrm{vol},0}\,, \tag{31}$$

$$\begin{aligned}\langle\sigma_{\mathrm{m}}(\mathbf{x})\rangle_{\mathrm{bone}} = {} & \frac{2E_{\mathrm{vol},0}\mathcal{F}^{-\frac{n_{\mathrm{bone}}}{3}}}{\mathcal{D}_k(\mathcal{F}-1)(4n^2_{\mathrm{bone}}-9)}\left\{\overline{\Gamma^k_{\mathrm{bone},1}}\left[\left(\sqrt{\mathcal{F}}-\mathcal{F}^{-\frac{n_{\mathrm{bone}}}{3}}\right)(3-2n_{\mathrm{bone}})\left(\mathcal{M}_{\mathrm{bone}}+2\mathcal{O}_{\mathrm{bone}}\right)\right]+\right.\\ & \left.+\overline{\Gamma^k_{\mathrm{bone},2}}\left[\left(\sqrt{\mathcal{F}}-\mathcal{F}^{\frac{n_{\mathrm{bone}}}{3}}\right)(3+2n_{\mathrm{bone}})\left(\mathcal{N}_{\mathrm{bone}}+2\mathcal{P}_{\mathrm{bone}}\right)\right]\right\}.\end{aligned} \tag{32}$$

3 DERIVATION OF THE MACROSCOPIC SHEAR MODULUS OF THE SCAFFOLD-BONE CONGLOMERATE

3.1 DERIVATION OF GOVERNING DIFFERENTIAL EQUATIONS

Substitution of the displacement field given by Eq. (35) of the main paper into the strain field, expressed in terms of spherical coordinates, and insertion of the strain field into the constitutive relations, Eqs. (12), Eqs. (13), and Eqs. (14) of the main paper, gives access, via the equilibrium condition, Eq. (5) of the main paper, to two ordinary differential equations,

$$\begin{aligned}&\frac{\mathrm{d}^2\xi_r}{\mathrm{d}r^2}+\frac{2}{r}\frac{\mathrm{d}\xi_r}{\mathrm{d}r}+\frac{\xi_r}{r^2}\frac{2(C_{i,rr\vartheta\vartheta}-3C_{i,r\vartheta r\vartheta}-C_{i,\vartheta\vartheta\varphi\varphi}-C_{i,\vartheta\vartheta\vartheta\vartheta})}{C_{i,rrrr}}-\\ &-\frac{1}{r}\frac{\mathrm{d}\xi_\vartheta}{\mathrm{d}r}\frac{3(C_{i,rr\vartheta\vartheta}+C_{i,r\vartheta r\vartheta})}{C_{i,rrrr}}+\frac{\xi_\vartheta}{r^2}\frac{3(C_{i,\vartheta\vartheta\vartheta\vartheta}-C_{i,rr\vartheta\vartheta}+C_{i,r\vartheta r\vartheta}+C_{i,\vartheta\vartheta\varphi\varphi})}{C_{i,rrrr}}=0\end{aligned} \tag{33}$$

$$\begin{aligned}&\frac{1}{r}\frac{\mathrm{d}\xi_r}{\mathrm{d}r}\frac{2(C_{i,rr\vartheta\vartheta}+C_{i,r\vartheta r\vartheta})}{C_{i,r\vartheta r\vartheta}}+\frac{\xi_r}{r^2}\frac{2(C_{i,r\vartheta r\vartheta}+C_{i,\vartheta\vartheta\varphi\varphi}+C_{i,\vartheta\vartheta\vartheta\vartheta})}{C_{i,r\vartheta r\vartheta}}+\\ &+\frac{\mathrm{d}^2\xi_\vartheta}{\mathrm{d}r^2}+\frac{2}{r}\frac{\mathrm{d}\xi_\vartheta}{\mathrm{d}r}-\frac{\xi_\vartheta}{r^2}\frac{2C_{i,r\vartheta r\vartheta}+C_{i,\vartheta\vartheta\varphi\varphi}+5C_{i,\vartheta\vartheta\vartheta\vartheta})}{C_{i,r\vartheta r\vartheta}}=0\,.\end{aligned} \tag{34}$$

The general solutions of Eqs. (33) and (34) are specified in the main paper; see Eqs. (33) – (35) of the main paper for anisotropic materials, and Eqs. (37) – (39) of the main paper for isotropic materials.

3.2 MATERIAL CONSTANTS

The general solution for the displacement field of an RVE subjected to simple shear, established via Eqs. (33) – (35) of the main paper, includes material functions $P_{i,11}$ and $P_{i,12}$, see Eq. (36) of the main paper. The mathematical basis for how these functions are derived is described at length elsewhere (Bertrand and Hellmich, 2009); they are defined as follows:

$$P_{i,11}(\alpha_{i,j}) = \left(\alpha^2_{i,j}-\frac{1}{4}\right)C_{i,rrrr}-6C_{i,r\vartheta r\vartheta}-2\left(C_{i,\vartheta\vartheta\varphi\varphi}-C_{i,rr\vartheta\vartheta}+C_{i,\vartheta\vartheta\vartheta\vartheta}\right)\,, \tag{35}$$

$$P_{i,12}(\alpha_{i,j}) = 3\left[\left(\frac{3}{2}+\alpha_{i,j}\right)C_{i,r\vartheta r\vartheta}-\left(\frac{1}{2}-\alpha_{i,j}\right)C_{i,rr\vartheta\vartheta}+C_{i,\vartheta\vartheta\vartheta\vartheta}+C_{i,\vartheta\vartheta\varphi\varphi}\right]\,, \tag{36}$$

where functions $\alpha_{i,j}$ are defined as

$$\alpha_{i,j} = \omega_j \sqrt{\frac{-L_{i,2} - \zeta_j \sqrt{L_{i,2}^2 - 4L_{i,0}L_{i,4}}}{2L_{i,4}}} \tag{37}$$

$$\text{with} \quad \omega_j = \begin{cases} -1 & \text{if } j = 1,2 \\ 1 & \text{if } j = 3,4 \end{cases} \quad \text{and} \quad \zeta_j = \begin{cases} 1 & \text{if } j = 1,4 \\ -1 & \text{if } j = 2,3\,. \end{cases}$$

Furthermore, coefficients $L_{i,0}$, $L_{i,2}$, and $L_{i,4}$ are solely governed by the components of the stiffness tensor of constituent i, expressed in spherical coordinates:

$$\begin{aligned} L_{i,0} = \frac{1}{16}\Big[&C_{i,rrrr}(C_{i,r\vartheta r\vartheta} + 4C_{i,\vartheta\vartheta\varphi\varphi} + 20C_{i,\vartheta\vartheta\vartheta\vartheta})+ \\ &+ 4C_{i,\vartheta\vartheta\vartheta\vartheta}(16C_{i,\vartheta\vartheta\vartheta\vartheta} + 66C_{i,r\vartheta r\vartheta}) + 8C_{i,rr\vartheta\vartheta}(9C_{i,r\vartheta r\vartheta} + 8C_{i,\vartheta\vartheta\varphi\varphi}- \\ &- 8C_{i,\vartheta\vartheta\vartheta\vartheta} - 3C_{i,rr\vartheta\vartheta}) - 8C_{i,\vartheta\vartheta\varphi\varphi}(15C_{i,r\vartheta r\vartheta} + 8C_{i,\vartheta\vartheta\varphi\varphi})\Big]\,, \end{aligned} \tag{38}$$

$$\begin{aligned} L_{i,2} = 2\Big[&C_{i,rr\vartheta\vartheta}(3C_{i,rr\vartheta\vartheta} + 7C_{i,r\vartheta r\vartheta}) - C_{i,r\vartheta r\vartheta}(C_{i,\vartheta\vartheta\varphi\varphi} + C_{i,\vartheta\vartheta\vartheta\vartheta})\Big] - \frac{1}{2}C_{i,rrrr}\times \\ &\times (5C_{i,r\vartheta r\vartheta} + 2C_{i,\vartheta\vartheta\varphi\varphi} + 10C_{i,\vartheta\vartheta\vartheta\vartheta})\,, \end{aligned} \tag{39}$$

$$L_{i,4} = C_{i,rrrr}C_{i,r\vartheta r\vartheta}\,. \tag{40}$$

3.3 STRAIN AND STRESS FIELDS FOR DEVIATORIC LOADING, AND THE GOVERNING COEFFICIENTS

The strain field corresponding to the displacement fields for simple shear, the latter being defined by Eqs. (33) – (35) of the main paper, follows through insertion of these equations into the kinematic relation, Eq. (10) of the main paper, specified for spherical coordinates, yielding

$$\varepsilon_{i,rr} = -\sum_{j=1}^{4} \frac{1}{2}(1 + 2\alpha_{i,j})\Gamma^{\mu}_{i,j} r^{-\frac{3}{2}-\alpha_{i,j}} \cos 2\varphi \sin^2\vartheta\,, \tag{41}$$

$$\varepsilon_{i,\vartheta\vartheta} = \sum_{j=1}^{4} \frac{1}{2}\Gamma^{\mu}_{i,j} r^{-\frac{3}{2}-\alpha_{i,j}} \cos 2\varphi\; [1 + (2\beta(\alpha_{i,j}) - 1)\cos 2\vartheta]\,, \tag{42}$$

$$\varepsilon_{i,\varphi\varphi} = \sum_{j=1}^{4} \Gamma^{\mu}_{i,j} r^{-\frac{3}{2}-\alpha_{i,j}} \cos 2\varphi\; \big[\beta(\alpha_{i,j}\,\big(\cos^2\vartheta - 2\big) + \sin^2\vartheta\big]\,, \tag{43}$$

$$\varepsilon_{i,r\vartheta} = -\sum_{j=1}^{4} \frac{1}{8}\Gamma^{\mu}_{i,j}\, [(3 + 2\alpha_{i,j})\,\beta(\alpha_{i,j}) - 4]\, r^{-\frac{3}{2}-\alpha_{i,j}} \cos 2\varphi \sin 2\vartheta\,, \tag{44}$$

$$\varepsilon_{i,\vartheta\varphi} = -\sum_{j=1}^{4} \Gamma^{\mu}_{i,j}\beta(\alpha_{i,j}) r^{-\frac{3}{2}-\alpha_{i,j}} \cos\vartheta \sin 2\varphi\,, \tag{45}$$

$$\varepsilon_{i,r\varphi} = \sum_{j=1}^{4} \frac{1}{4}\Gamma^{\mu}_{i,j}\, [(3 + 2\alpha_{i,j})\,\beta(\alpha_{i,j}) - 4]\, r^{-\frac{3}{2}-\alpha_{i,j}} \sin 2\varphi \sin\vartheta\,. \tag{46}$$

Insertion of the strain tensor components, given by Eqs. (41) – (46), into the constitutive relations, Eqs. (12), Eqs. (13), and Eqs. (14) of the main paper, gives access to the corresponding stress field:

$$\sigma_{i,rr} = -\sum_{j=1}^{4} \frac{1}{2}\Gamma^{\mu}_{i,j} \left[C_{i,rrrr}(1+2\alpha_{i,j}) - 4C_{i,rr\vartheta\vartheta} + 6\beta(\alpha_{i,j})C_{i,rr\vartheta\vartheta}\right] r^{-\frac{3}{2}-\alpha_{i,j}} \cos 2\varphi \, \sin^2\vartheta \,, \tag{47}$$

$$\begin{aligned}\sigma_{i,\vartheta\vartheta} = &\sum_{j=1}^{4} \frac{1}{2}\Gamma^{\mu}_{i,j} r^{-\frac{3}{2}-\alpha_{i,j}} \Big\{ \left[1 + (1-2\beta(\alpha_{i,j}))\,\cos 2\vartheta\right] C_{i,\vartheta\vartheta\vartheta\vartheta} - \\ &- 2\left(2\beta(\alpha_{i,j}) - \cos^2\vartheta\right) C_{i,\vartheta\vartheta\varphi\varphi} - \left(C_{i,rr\vartheta\vartheta} + 2\alpha_{i,j}C_{i,rr\vartheta\vartheta} - 2C_{i,\vartheta\vartheta\varphi\varphi}\right)\sin^2\vartheta \Big\} \,, \end{aligned}\tag{48}$$

$$\begin{aligned}\sigma_{i,\varphi\varphi} = &\sum_{j=1}^{4} \frac{1}{2}\Gamma^{\mu}_{i,j} r^{-\frac{3}{2}-\alpha_{i,j}} \Big\{ 2\beta(\alpha_{i,j})\left(\cos^2\vartheta - 2\right) C_{i,\vartheta\vartheta\vartheta\vartheta} + \left[1 + (2\beta(\alpha_{i,j}) - 1)\cos 2\vartheta\right] \times \\ &\times C_{i,\vartheta\vartheta\varphi\varphi} - \left((1+2\alpha_{i,j})\, C_{i,rr\vartheta\vartheta} - 2C_{i,\vartheta\vartheta\vartheta\vartheta}\right)\sin^2\vartheta \Big\} \cos 2\varphi \,, \end{aligned}\tag{49}$$

$$\sigma_{i,r\vartheta} = \sum_{j=1}^{4} \Gamma^{\mu}_{i,j}\beta(\alpha_{i,j})(C_{i,\vartheta\vartheta\varphi\varphi} - C_{i,\vartheta\vartheta\vartheta\vartheta}) r^{-\frac{3}{2}-\alpha_{i,j}} \cos\vartheta \sin 2\varphi \,, \tag{50}$$

$$\sigma_{i,\vartheta\varphi} = \sum_{j=1}^{4} \Gamma^{\mu}_{i,j} \left[(3+2\alpha_{i,j})\beta(\alpha_{i,j}) - 4\right] C_{i,r\vartheta r\vartheta} r^{-\frac{3}{2}-\alpha_{i,j}} \cos\varphi \, \sin\varphi \, \sin\vartheta \,, \tag{51}$$

$$\sigma_{i,r\varphi} = -\sum_{j=1}^{4} \frac{1}{4}\Gamma^{\mu}_{i,j} \left[(3+2\alpha_{i,j})\,\beta(\alpha_{i,j}) - 4\right] C_{i,r\vartheta r\vartheta} r^{-\frac{3}{2}-\alpha_{i,j}} \cos 2\varphi \, \sin 2\vartheta \,. \tag{52}$$

For determination of the twelve parameters $\Gamma^{\mu}_{i,j}$ ($i =$ glob, bone, scaff; $j = 1, 2, 3, 4$), one may start with evaluation of Eq. (37) [or Eq. (38)] of the main paper, for $r = 0$: $\Gamma^{\mu}_{\text{gran},3} = 0$ and $\Gamma^{\mu}_{\text{gran},4} = 0$. Furthermore, the requirement of finite displacements at $r \to \infty$ implies $\Gamma^{\mu}_{\text{congl},1} = \gamma$ and $\Gamma^{\mu}_{\text{congl},2} = 0$. The remaining eight parameters are again obtained based on continuity equations formulated at the interfaces between the different domains of which the RVE is composed,

$$\xi_{\text{gran},r}(r_1) = \xi_{\text{bone},r}(r_1), \tag{53}$$

$$\xi_{\text{gran},\vartheta}(r_1) = \xi_{\text{bone},\vartheta}(r_1), \tag{54}$$

$$\xi_{\text{bone},r}(r_2) = \xi_{\text{congl},r}(r_2), \tag{55}$$

$$\xi_{\text{bone},\vartheta}(r_2) = \xi_{\text{congl},\vartheta}(r_2), \tag{56}$$

$$\sigma_{\text{gran},rr}(r_1) = \sigma_{\text{bone},rr}(r_1), \tag{57}$$

$$\sigma_{\text{gran},r\vartheta}(r_1) = \sigma_{\text{bone},r\vartheta}(r_1), \tag{58}$$

$$\sigma_{\text{bone},rr}(r_2) = \sigma_{\text{congl},rr}(r_2), \tag{59}$$

$$\sigma_{\text{bone},r\vartheta}(r_2) = \sigma_{\text{congl},r\vartheta}(r_2) \,. \tag{60}$$

Based on insertion of the respective displacement and stress components into Eqs. (53) – (60) eventually yields a system of eight linear equations. Solution of this system of equations, here performed as suggested

by Hervé and Zaoui (1993), gives access to

$$\Gamma^{\mu}_{\text{gran},1} = \gamma\overline{\Gamma^{\mu}_{\text{gran},1}}\,, \tag{61}$$

$$\Gamma^{\mu}_{\text{gran},2} = \gamma\overline{\Gamma^{\mu}_{\text{gran},2}}r_1^{-2}\,, \tag{62}$$

$$\Gamma^{\mu}_{\text{bone},j} = \gamma\overline{\Gamma^{\mu}_{\text{bone},j}}r_1^{\frac{3}{2}+\alpha_{\text{bone},j}}\,, \tag{63}$$

$$\Gamma^{\mu}_{\text{congl},3} = \gamma\overline{\Gamma^{\mu}_{\text{congl},3}}r_1^{5}\,, \tag{64}$$

$$\Gamma^{\mu}_{\text{congl},4} = \gamma\overline{\Gamma^{\mu}_{\text{congl},4}}r_1^{3}\,, \tag{65}$$

with Eq. (63) being valid for $j = 1,2,3,4$. The overlined quantities in Eqs. (61) – (65), that is $\overline{\Gamma^{\mu}_{\text{gran},1}}$, $\overline{\Gamma^{\mu}_{\text{gran},2}}$, $\overline{\Gamma^{\mu}_{\text{bone},j}}$ $(j = 1,2,3,4)$, $\overline{\Gamma^{\mu}_{\text{congl},3}}$, and $\overline{\Gamma^{\mu}_{\text{congl},4}}$, are governed by phase volume fractions and phase stiffness tensors via lengthy expressions. For this reason, we refrain from explicitly presenting these expressions here.

3.4 VOLUME AVERAGES OF STRAIN AND STRESS FIELDS IN GRANULE AND BONE PHASES FOR DEVIATORIC LOADING

Application of Eq. (54) of the main paper to the granule and the bone phases yields

$$\big\langle \boldsymbol{\varepsilon}_{\text{d}}(\mathbf{x})\big\rangle_{\text{gran}} = \left(\overline{\Gamma^{\mu}_{\text{gran},1}} - \frac{21}{5}\frac{\overline{\Gamma^{\mu}_{\text{gran},2}}}{1-2\nu_{\text{gran}}}\right)\mathbf{E}_{\text{d},0}\,, \tag{66}$$

$$\big\langle \boldsymbol{\varepsilon}_{\text{d}}(\mathbf{x})\big\rangle_{\text{bone}} = \frac{1}{5}\left[\sum_{j=1}^{4}\frac{1-\mathcal{F}^{\frac{1}{2}-\frac{\alpha_{\text{bone},j}}{3}}}{1-\mathcal{F}}\overline{\Gamma^{\mu}_{\text{bone},j}}\left[2+3\beta(\alpha_{\text{bone},j})\right]\right]\mathbf{E}_{\text{d},0}\,. \tag{67}$$

Furthermore, application of Eq. (55) of the main paper to the granule and the bone phase yields

$$\big\langle \boldsymbol{\sigma}_{\text{d}}(\mathbf{x})\big\rangle_{\text{gran}} = 2\mu_{\text{gran}}\left(\overline{\Gamma^{\mu}_{\text{gran},1}} - \frac{21}{5}\frac{\overline{\Gamma^{\mu}_{\text{gran},2}}}{1-2\nu_{\text{gran}}}\right)\mathbf{E}_{\text{d},0}\,, \tag{68}$$

$$\big\langle \boldsymbol{\sigma}_{\text{d}}(\mathbf{x})\big\rangle_{\text{bone}} = \left[\sum_{j=1}^{4}\frac{1-\mathcal{F}^{\frac{1}{2}-\frac{\alpha_{\text{bone},j}}{3}}}{1-\mathcal{F}}\overline{\Gamma^{\mu}_{\text{bone},j}}\kappa(\alpha_{\text{bone},j})\right]\mathbf{E}_{\text{d},0}\,, \tag{69}$$

with $\kappa(\alpha_{\text{bone},j})$ as additional material parameter, defined as follows:

$$\begin{aligned}\kappa(\alpha_{\text{bone},j}) = \frac{2}{5(-3+2\alpha_{\text{bone},j})}\Big\{&C_{\text{bone},rrrr} + 2\alpha_{\text{bone},j}C_{\text{bone},rrrr} -\\ &-\left[5+2\alpha_{\text{bone},j}-6\beta(\alpha_{\text{bone},j})\right]C_{\text{bone},rr\vartheta\vartheta} +\\ &+3\beta(\alpha_{\text{bone},j})\left[(3+2\alpha_{\text{bone},j})C_{\text{bone},r\vartheta r\vartheta} + C_{\text{bone},\vartheta\vartheta\varphi\varphi} - 3C_{\text{bone},\vartheta\vartheta\vartheta\vartheta}\right] +\\ &+2(C_{\text{bone},\vartheta\vartheta\varphi\varphi} + C_{\text{bone},\vartheta\vartheta\vartheta\vartheta} - 6C_{\text{bone},r\vartheta r\vartheta})\Big\}\,.\end{aligned} \tag{70}$$

REFERENCES

Bertrand, E. and Hellmich, C. (2009). Multiscale elasticity of tissue engineering scaffolds with tissue-engineered bone: a continuum micromechanics approach. *Journal of Engineering Mechanics (ASCE)*, 135(5):395–412.

Eshelby, J. (1957). The determination of the elastic field of an ellipsoidal inclusion, and related problems. *Proceedings of the Royal Society London, Series A*, 241:376–396.

Fritsch, A. and Hellmich, C. (2007). 'Universal' microstructural patterns in cortical and trabecular, extracellular and extravascular bone materials: micromechanics-based prediction of anisotropic elasticity. *Journal of Theoretical Biology*, 244(4):597–620.

Hellmich, C., Barthélémy, J.-F., and Dormieux, L. (2004). Mineral-collagen interactions in elasticity of bone ultrastructure - a continuum micromechanics approach. *European Journal of Mechanics - A/Solids*, 23(5):783 – 810.

Hervé, E. and Zaoui, A. (1993). n-Layered inclusion-based micromechanical modelling. *International Journal of Engineering Science*, 31(1):1–10.

Laws, N. (1977). The determination of stress and strain concentrations at an ellipsoidal inclusion in an anisotropic material. *Journal of Elasticity*, 7(1):91 – 97.

Laws, N. (1985). A note on penny-shaped cracks in transversely isotropic materials. *Mechanics of Materials*, 4(2):209 – 212.

Zaoui, A. (2002). Continuum micromechanics: survey. *Journal of Engineering Mechanics (ASCE)*, 128(8):808–816.
